# The NERVE-ML (neural engineering reproducibility and validity essentials for machine learning) checklist: ensuring machine learning advances neural engineering^*^

**DOI:** 10.1088/1741-2552/adbfbd

**Published:** 2025-03-27

**Authors:** David E Carlson, Ricardo Chavarriaga, Yiling Liu, Fabien Lotte, Bao-Liang Lu

**Affiliations:** 1 Department of Biostatistics and Bioinformatics, Duke University School of Medicine, Durham, NC, United States of America; 2 Department of Computer Science, Department of Civil and Environmental Engineering, Duke University, Durham, NC, United States of America; 3 Centre for Artificial Intelligence, School of Engineering, Zurich University of Applied Sciences ZHAW, Winterthur, Switzerland; 4 Program in Computational Biology and Bioinformatics, Duke University School of Medicine, Durham, NC, United States of America; 5 Inria Center at the University of Bordeaux, Talence 33405, France; 6 LaBRI (CNRS/University Bordeaux/Bordeaux INP), Talence 33405, France; 7 Center for Brain-Like Computing and Machine Intelligence, Department of Computer Science and Engineering, Shanghai Jiao Tong University, Shanghai 200240, People’s Republic of China; 8 RuiJin-Mihoyo Laboratory, Clinical Neuroscience Center, RuiJin Hospital, Shanghai Jiao Tong University School of Medicine, Shanghai 200020, People’s Republic of China

**Keywords:** machine learning, reproducibility, validation, checklist

## Abstract

*Objective.* Machine learning’s (MLs) ability to capture intricate patterns makes it vital in neural engineering research. With its increasing use, ensuring the validity and reproducibility of ML methods is critical. Unfortunately, this has not always been the case in practice, as there have been recent retractions across various scientific fields due to the misuse of ML methods and validation procedures. To address these concerns, we propose the first version of the neural engineering reproducibility and validity essentials for ML (NERVE-ML) checklist, a framework designed to promote the transparent, reproducible, and valid application of ML in neural engineering. *Approach.* We highlight some of the unique challenges of model validation in neural engineering, including the difficulties from limited subject numbers, repeated or non-independent samples, and high subject heterogeneity. Through detailed case studies, we demonstrate how different validation approaches can lead to divergent scientific conclusions, highlighting the importance of selecting appropriate procedures guided by the NERVE-ML checklist. Effectively addressing these challenges and properly scoping scientific conclusions will ensure that ML contributes to, rather than hinders, progress in neural engineering. *Main results.* Our case studies demonstrate that improper validation approaches can result in flawed studies or overclaimed scientific conclusions, complicating the scientific discourse. The NERVE-ML checklist effectively addresses these concerns by providing guidelines to ensure that ML approaches in neural engineering are reproducible and lead to valid scientific conclusions. *Significance.* By effectively addressing these challenges and properly scoping scientific conclusions guided by the NERVE-ML checklist, we aim to help pave the way for a future where ML reliably enhances the quality and impact of neural engineering research.

## 1. Introduction

The use of artificial intelligence (AI) through machine learning (ML) is now a critical tool in neural engineering, mainly because of its ability to capture complex patterns in neural data more flexibly than traditional data analysis methods. Its popularity and usage continue to grow each year, as measured by the number of articles mentioning ML^9^9For example, at the Journal of Neural Engineering, 60% of all articles retrieved from a search on ‘machine learning’ had been published in the last 5 years (accessed 27 September 2024).. As ML becomes increasingly integrated into neural engineering, it is crucial to develop and enforce high standards for research validity and reproducibility in ML models to ensure that these techniques improve and accelerate the field.

Incorrect application of ML can lead to wrong conclusions, setting back scientific progress and potentially leading to incorrect follow-up research or worse, including failed attempts to translate these findings into applications. While the computational methods themselves have changed, this fact is a natural extension of the long-standing challenges of incorrect statistical conclusions [1] and the prevalence of unreproducible results [2]. This problem is highlighted by the number of papers retracted due to errors in the ML validation procedure, from data handling mistakes to severe biases in data, algorithms or evaluation procedures. Additionally, many papers with incorrect conclusions have not been withdrawn, and such papers cloud the scientific record [3].

For the sake of rigorous science, the use of ML in neural engineering research must be transparent, reproducible, and properly scoped. The scientific conclusions drawn from these models need to be valid. Limitations need to be understood and disclosed. Upholding these principles will ensure quality research and build trust in ML within the broad scientific community. This parallels efforts more broadly in society, where trustworthy AI is needed to promote positive societal impact [4, 5], as evidenced by the European efforts of the General Data Protection Regulation and the AI Act and the United States’ efforts in the Blueprint for an AI Bill of Rights.

There have been numerous studies and reviews on using ML in neuroscience and neural engineering [6, 7]. We complement this body of work by providing guidelines that focus on how to validate the use of ML to ensure reproducibility and validity in neural engineering research. Similar challenges are currently being addressed in a wide variety of disciplines. This effort aligns with initiatives in the ML community to create guidelines to address these issues [8], which have been adopted by many ML venues, including Neural Information Processing Systems (NeurIPS) and the International Conference on ML, among others. Likewise, there have been significant efforts to create checklists to encourage consideration of reproducibility of results in predictive models in medicine [9, 10]. There are several ongoing efforts to adapt such checklists to the particular use of AI and ML models [11]. However, neural engineering presents unique challenges, such as working with a small number of subjects and data, variability in devices, possible lack of statistical independence between samples and differences between individuals. These unique challenges require that we tailor the standard ML guidelines to neural engineering, as proposed here.

Our goal is to ensure that ML positively impacts neural engineering by *providing a robust framework for study scoping and interpretation*. We first provide details on ML practices and how inappropriately handling some of these challenges can cause incorrect interpretations through case studies and simulations. We then suggest best practices and introduce the neural engineering reproducibility and validity essentials for ML (NERVE-ML) checklist. This checklist aims to guide researchers in ensuring their ML research in neural engineering is reproducible and the conclusions are valid. While it does not cover every situation, we believe that this is a step towards ensuring reliable ML.

Our effort complements other ongoing and recent initiatives within the field of neural engineering. For instance, the Mother of All BCI Benchmarks (MOABB) [12–14] provides a standardized framework for benchmarking ML algorithms specifically within brain–computer interface (BCI) research, emphasizing reproducibility and cross-dataset comparability. Additionally, Roy *et al* [2] provide a systematic review of deep learning (DL) methods for electroencephalography (EEG) analysis, highlighting variability in validation practices and reporting. They propose a DL-EEG checklist to help with reporting key algorithmic details, which shares some overlap with our proposed checklist.

In contrast, our work specifically addresses validation procedures and their direct influence on scientific conclusions. Proper validation is critical for ensuring reproducible results, preventing inflated performance estimates, and avoiding misinterpretation of findings. Note that a recent survey that found that many clinical neural engineering papers using EEG and DL for medical diagnosis were incorrectly splitting their training and testing data [15]. While MOABB and similar initiatives provide valuable benchmarks for ML models, they do not offer generalizable frameworks for addressing the diverse challenges inherent in validating performance on neural engineering datasets. Our checklist fills this gap by offering a systematic approach to link validation strategies with scientific objectives, ensuring that research in neural engineering is reproducible, rigorous, and meaningful.

The rest of this paper is structured as follows. Prior to introducing our checklist, we begin with a review and tutorial of standard validation practices in section 2. In this section, we will consider real-world examples in neural engineering and illustrate the effect of applying different validation techniques to real-world data. We will show how common validation errors lead to overstated performance and incorrect scientific conclusions. Then, section 3 discusses other considerations for ML beyond predictive performance. This highlights that ML methods do not exist in a vacuum. They are part of a broader ecosystem of use, and their utility depends on how they are designed and used by both practitioners and (potentially non-expert) target users. Section 4 presents the NERVE-ML checklist in detail, highlighting its tailored approach to addressing the unique challenges of neural engineering, such as reproducibility, validation, and translational impact. Section 5 concludes our discussion.

### 2. Validation and model selection

**Figure 1.**
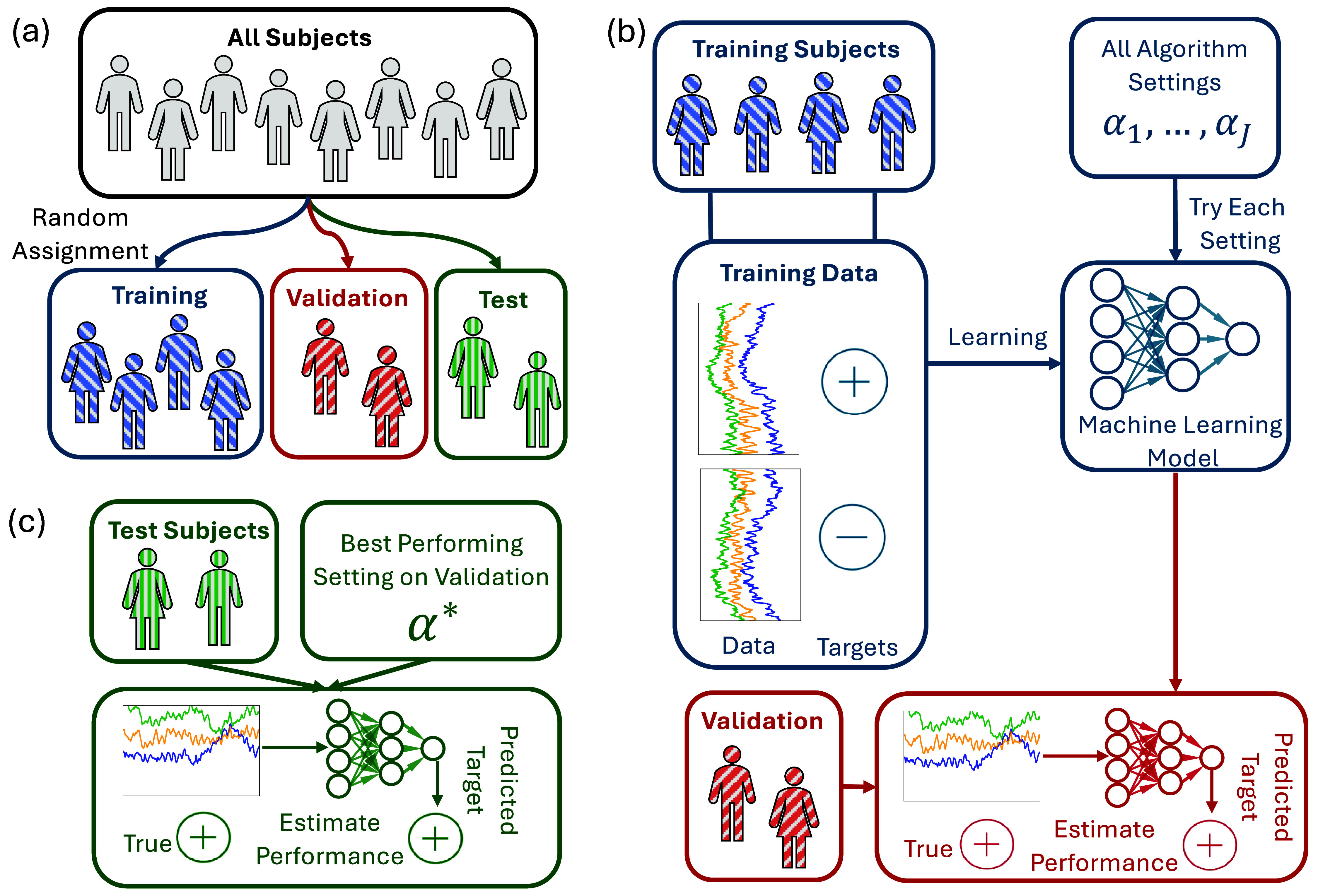
Overview of a typical machine learning pipeline. (a) All subjects are randomly split into train, validation, and test data as described in the text. Blue, red, and green denote the training, validation, and test process, respectively. (b) All training subjects have training data, which may comprise of either a single datum or multiple instances for each subject along with known targets in supervised machine learning. For each possible algorithm setting (e.g. type and shape of neural network, penalization strength, etc), the machine learning method (or algorithm) is trained or learned from the training data and targets. After the algorithm has been learned, it is evaluated on the validation data to estimate performance on data unseen by the training approach. (c) The best performing algorithm is chosen based on estimated performance on the validation data. As that algorithm is chosen based on the best validation performance, it is a biased estimate due to the many comparisons. Thus, for that single chosen algorithm, it is evaluated on the test data to give a more reliable estimate of performance.

The validation process attempts to answer the question: how well will a ML system trained on a subset of data generalize to the entire population? The canonical approach in a data-rich scenario is to randomly split all data samples into training, validation, and test sets prior to analysis [16], which is shown in figure 1(a) where the data (samples corresponding to subjects or measurements) are randomly assigned to these sets. This approach is commonly used in large-scale datasets in DL, such as in the ImageNet challenge that helped advance DL techniques [17]; however, it is not always feasible in neural engineering, as we will highlight below. To briefly describe our nomenclature, the training data will be used to train ML models and learn the parameters in the ML system or model, the validation data will be used repeatedly to estimate performance of many models and perform model selection (meaning choosing which hyperparameters and/or model to use in practice), and the test data will be used only once to estimate population level performance. We note that the nomenclature is inconsistent in the ML literature, such as where ‘test’ and ‘validation’ have opposite meanings in different papers, or terms such as ‘holdout’, ‘blind’, ‘internal validation’, and ‘external validation’ are used in place of either of these terms. This lack of standard nomenclature and overlapping jargon creates some challenges and hinders reproducibility, so we will note when multiple terms are commonly used for the same idea.

Prior to deploying a system relying on a ML model, we need to assess whether its performance is fit for the intended purpose. The *validation* procedure mimics the deployment process of the ML model on a new real-world experiment by applying it to data previously unseen by the learning algorithm (i.e. different than the training data, and independent from it). In other words, we are trying to see how well the model generalizes to new, previously unseen data. Theoretically, trying a ML model on such new data can provide a good estimation of real-world performance.

Therefore, the validation procedure also plays a critical role in selecting the best model (‘model selection’) and hyperparameter tuning. It is generally not helpful to use classification performance on the training set to select models and hyperparameters. In fact, neural network methods often make zero training errors in classification problems [18]. This does not mean that they will perform equally well in the entire population. As such, we want to pick the best ML model and settings (or hyperparameters) by trying many possibilities and choosing the approach that will work the best on the true population. As the true population is inaccessible, we approximately find the optimal modeling approach by choosing the one with the highest performance on the validation data, which again is not used to train or fit the model. This procedure is shown in figure 1(b). Because we try many different approaches on the validation data and there is randomness due to the sample size, our validation performance estimates become biased [19]. In other words, if we try enough methods, one of them will appear better than it actually is by random chance. Therefore, we need to use the previously unused test data to get an unbiased estimate of population-level performance, or what is often called generalization error. That approach ensures that no test data is ever used to optimize the model (i.e. by tuning of its parameters or hyperparameters). This testing approach is shown in figure 1(c).

This data splitting scenario is simple and works well when data samples are truly independent and plentiful. However, it is often inappropriate for neural engineering, as it is in many fields. We focus here on three main issues. First, how we split the data into training, validation, and test can change what scientific question we ask of the data depending on its structure. This is especially important as data samples in neural engineering are seldom completely independent, such as multiple samples or sessions from a single individual. Second, it is rare to be in a truly data-rich scenario in neural engineering, and alternative methods, described below, can be beneficial for estimating population-level performance by reducing uncertainty and making better use of all data. Third, there are potential mismatches between the research data and the true population, which is not a unique problem in ML.

### 2.1. Validation with limited data

**Figure 2.**
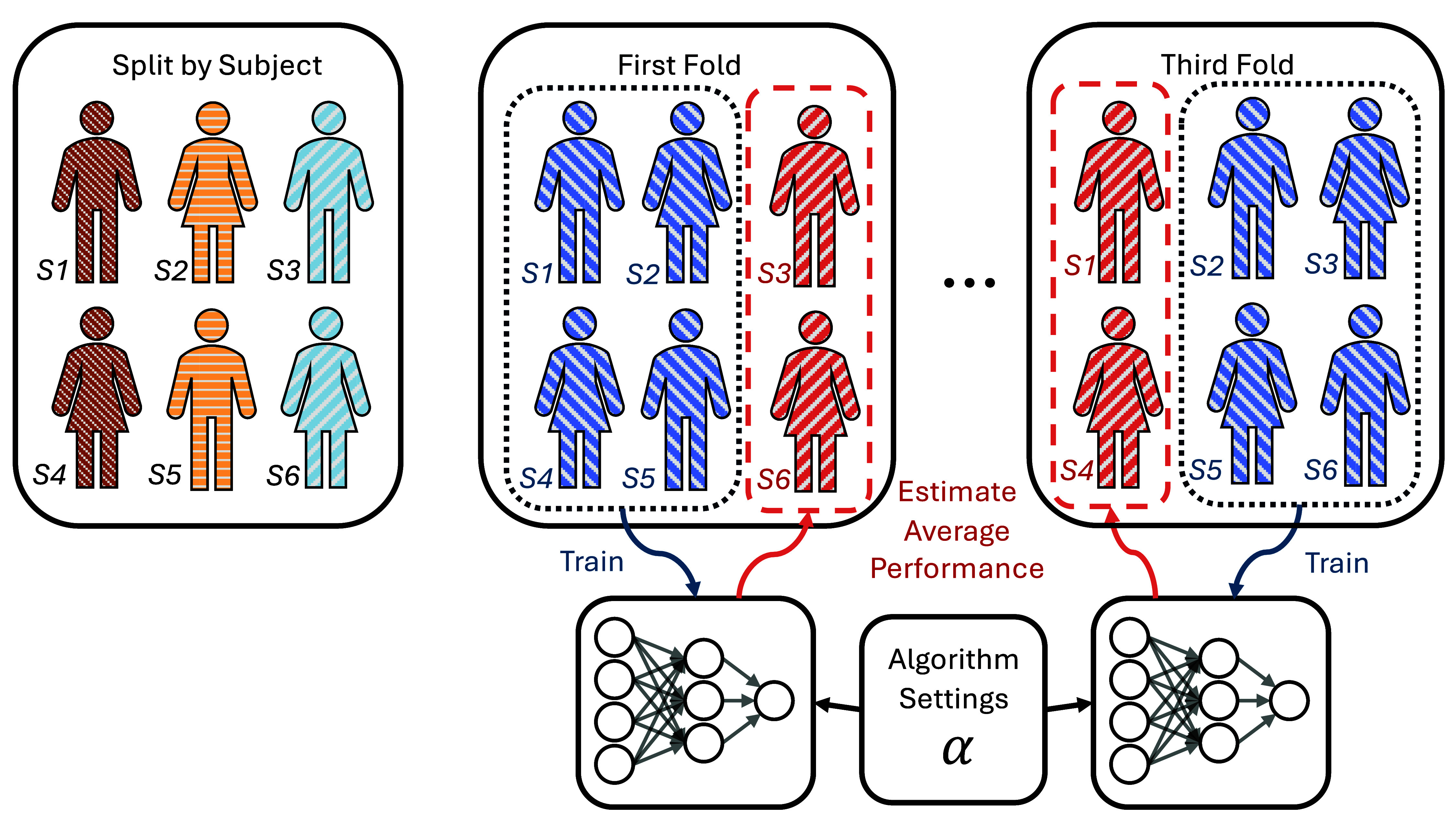
Visualization of cross-validation procedure. On the left, we show 6 subjects, each labeled with a subject ID (S1, S2, …). For a 3-fold cross validation, there are 3 different groups of data, and each subject is assigned to a single group. For each fold, 2 of groups are used as training data, and the remaining group is used as validation data. The training data is used to train the machine learning method, and the validation data is used to estimate performance. The performance is averaged over all folds to get a more reliable performance estimate for the chosen algorithm settings.

Neural engineering research is heavily limited by number of subjects and practical limits on data collection. As such, it is important to consider how we can use more of the data for validation and model selection. The most standard approach for this is to use cross-validation [16]. Rather than using a predefined training and validation set, the idea of cross-validation is that we create a pool of combined data that is repeatedly used to create training and validation data. The most common approach is to use $K$-fold cross-validation, where the data is split into $K$ non-overlapping validation sets. For example, if $K = $ 3, then three separate validation sets are formed. In each iteration, one of these sets is held out for validation while the remaining two are combined for training. After the model is trained on these two sets, its performance is ideally evaluated on the held-out data. This process is repeated $K$ times, ensuring that each of the $K$ subsets has served as a validation set. This process is visualized in figure 2. The advantage of *K*-fold cross-validation in neural engineering, with its limited data, is that it allows for more extensive use of the available data for both training and validation, thereby increasing the reliability of the validation process. Furthermore, it provides insights into the variability of model performance across different data subsets, highlighting potential biases or inconsistencies in the dataset.

There are multiple methods to get a final estimate of model performance. If cross-validation is run with test data held out, it means that a portion of the data is set aside right from the beginning and is not involved in the cross-validation process. This reserved test set would then ensure an unbiased assessment of model performance on unseen data. Such a strategy boosts confidence in the model’s generalization capabilities since the final evaluation is carried out on data that had no influence on the model’s training or hyperparameter tuning. However, this can be challenging in practice, as a 15% validation sample on 20 subjects is only 3 subjects. Given the typically large inter-subject variability in neural engineering, performance estimates will be highly uncertain. Such small numbers prohibit confidence in any performance numbers.

On the other hand, if cross-validation is run on all data, every data point is used in both the training and validation sets across different folds. While this approach maximizes the utilization of available data and may provide a more comprehensive view of model stability and performance variability, it lacks a final evaluation on an unseen dataset. Hence, the predictive performance tends to be overreported, although methods such as nested cross-validation can mitigate this bias [20, 21]. Overinflated performances with cross-validation are often observed in neural engineering [22]. For instance, neural signals are known to be highly variable and non-stationary across time. However, with cross-validation, it is not uncommon that the training data includes some samples collected before (in time) those of the validation data, and some collected after it. As such, the training data can capture this variability, contrary to a real online neural engineering experiments in which the test set is always later in time than the training set. Moreover, performance estimates with cross-validation in neural engineering with small data can lead to unstable performance estimates with large error bars [23]. Thus, especially in applications where deployment decisions are crucial, having an independent test set can be a safeguard against potential overfitting or other biases that might not be captured during the cross-validation process.

### 2.2. Statistical questions and data splitting

In this section, we explore how different data splitting procedures relate to different scientific questions. Ideally, our research question will determine how we split the data, as the choice of splitting strategy directly influences the validity and interpretability of the results. The goal of this section is to clarify how variations in data splitting strategies impact the scientific conclusions we draw from ML experiments. Different splitting strategies, such as dividing data by subjects, sessions, or randomly across the dataset, are all valid but serve distinct purposes and should be aligned with the specific scientific objectives. By carefully selecting the splitting method, we can ensure that the evaluation metrics meaningfully address the research question at hand.

This discussion builds on prior work emphasizing the importance of validation practices in neural data analysis. For instance, Varoquaux *et al* [23] highlight the biases that can arise from inappropriate cross-validation strategies, while Roy *et al* [2] point out how mismatched validation approaches can lead to inflated performance metrics in EEG-based ML studies. Our focus extends these insights by explicitly linking data splitting choices to the validity and interpretability of scientific conclusions in neural engineering.

**Figure 3.**
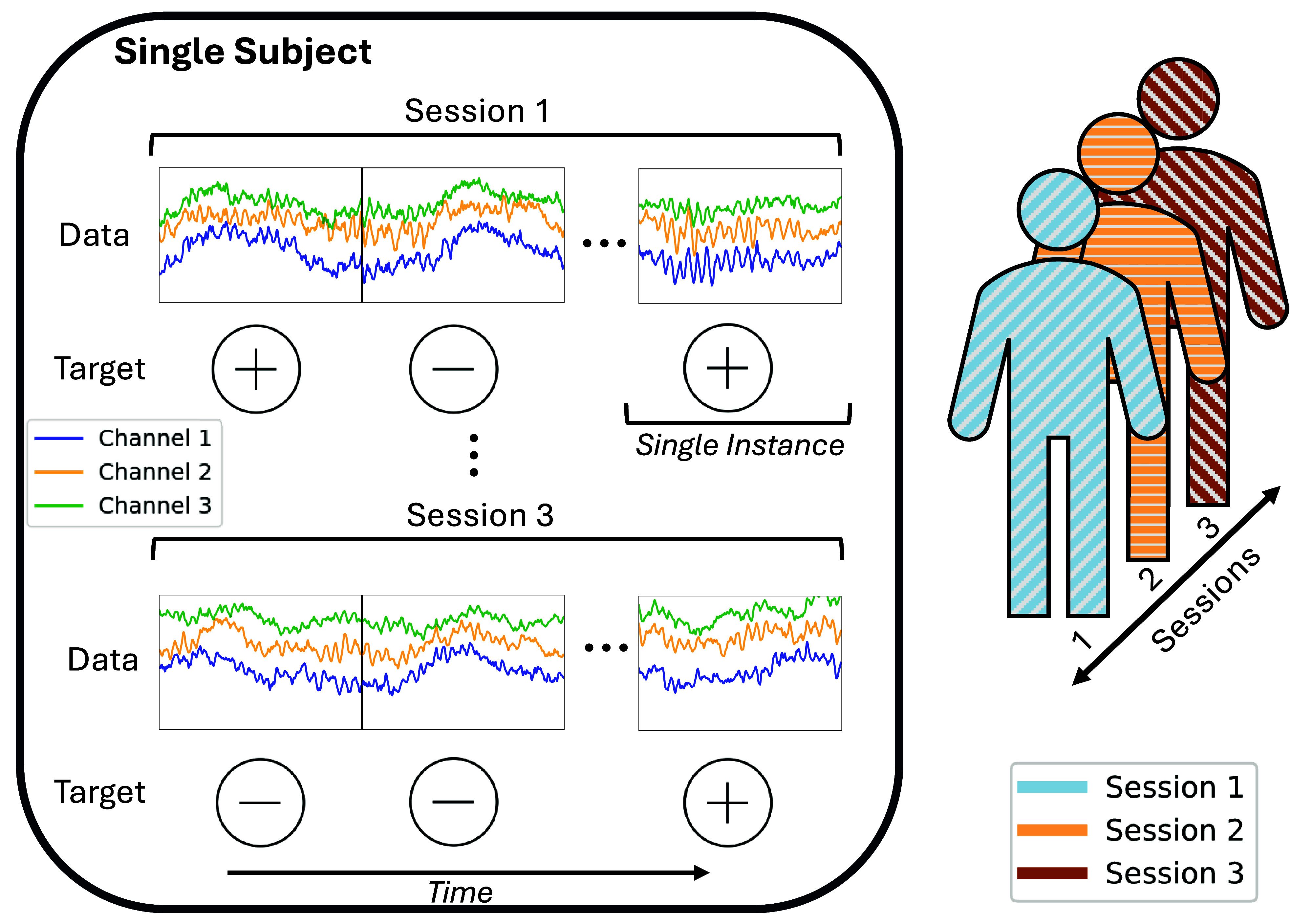
Visualization of data structure. Here, we visualize a single individual that has data collected over multiple sessions. For simplicity, we show a 3-channel temporal signal such as would be collected by an electrophysiological experiment, but many different types of data could be used instead. Each session has a separate start and stop point and is split into multiple instances or epochs and associated with a target. On the right, we show a representation of a single individual over the sessions, which is used to describe splitting strategies in figure 4.

As a concrete example, consider a tool where we classify brain data on a second-by-second basis, as is a common task in seizure detection, BCIs and emotion recognition. We assume that we have data from $M$ subjects all from the same medical center, and each subject $m$ has data split into ${N_m}$ instances (e.g. one instance for each second) with features $x_{mi}$ and targets $y_{mi}$^10^10The terms used for the features and targets are not standardized. It is common to instead call the features ‘predictors’, ‘covariates’, or ‘input variables’, among others. Likewise, it is common to call the targets ‘outcome’, ‘label’, ‘class’, or ‘dependent variable’, among others.. These data may have been collected over multiple sessions. A visualization of this data structure for a single subject is shown in figure 3, which shows a visualization of individual instances and sessions. We note that figure 3 uses multi-channel electrophysiological data and binary targets as an example, but the concept generalizes to many different data types and targets.

To understand how different splitting approaches can relate to different corresponding scientific questions, consider the concrete example of seizure detection. We may want to ask:

a. How well does the ML model predict targets on new instances from the same subject?
b. How well does the ML model predict targets on a new session (e.g. another day) from the same subject?
c. How well does the ML model predict targets on new subjects at the same medical center?
d. How well does the ML model predict targets on new subjects at a new medical center?

**Figure 4.**
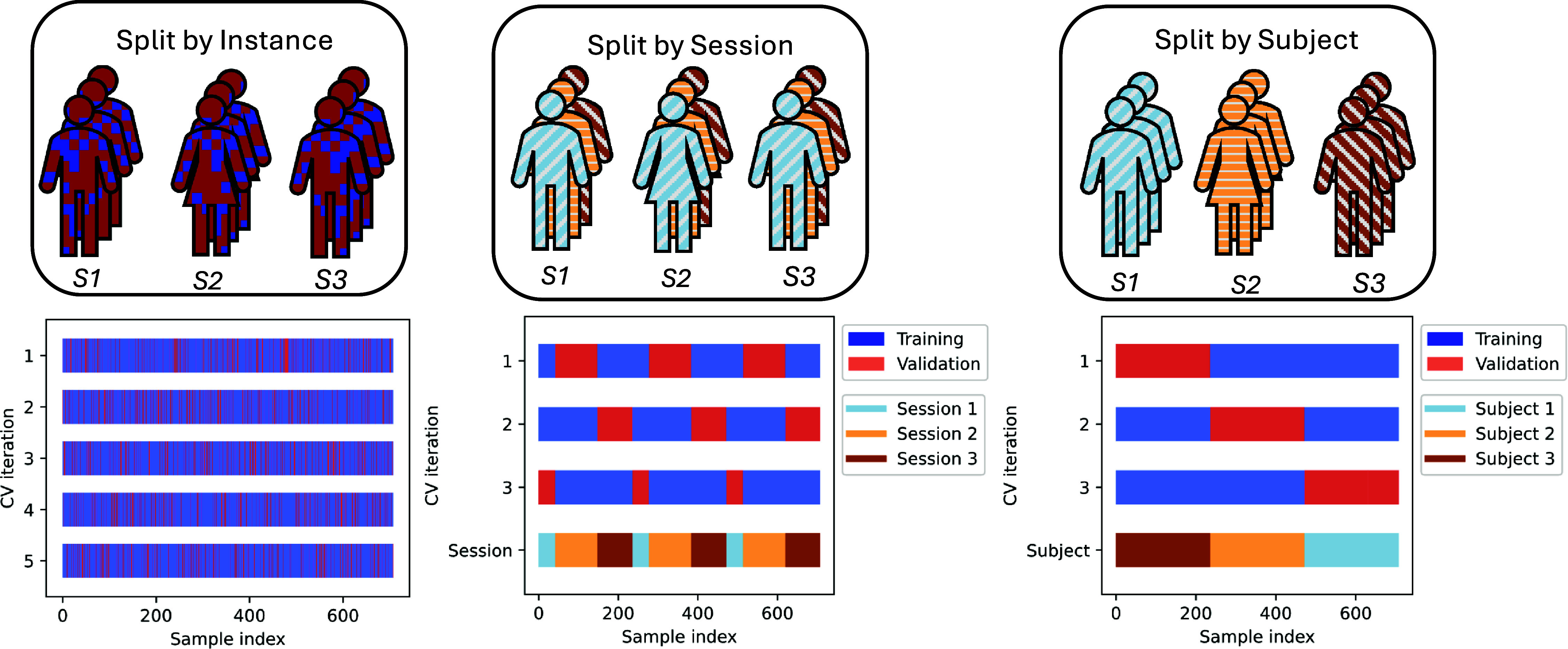
Visualization of different types of data splitting. In an experiment where there are repeated samples from subjects, the data splitting changes the statistical question being asked and estimated performance. Three different types of cross-validation procedures are shown on 3 subjects for visual clarity, each marked with a subject ID (S1, S2, S3). In practice, this process would happen on many more subjects. (a) Split by instance. The data is split so that each individual data instance is split randomly into the training (blue) or test/validation (red) data. Each subject will have some data in the training and test data without regards to the session structure. This is visualized with 2 folds for visual clarity but can be done with any number of folds. (b) Split by session. Here, each subject is split so that each session is in a different cross-validation fold. Each subject has some data in each fold, but only with regards to each session. The training may proceed on either each subject, or on all subjects simultaneously depending on the statistical question. (c) Split by subject. Each subject’s data is completely in a single cross-validation fold, so a subject is either completely in a training or validation data for each training loop.

The appropriate data splitting strategy is determined by the scientific research question. As highlighted above, the validation strategy should mimic a new real-world experiment aligned with that question. To answer question (a) with an experiment, we would collect more data from the same subject during the same session, and then evaluate the performance on these newly acquired instances. This can be mimicked in a validation procedure by randomly choosing instances, and then evaluating on previously unseen instances. This is visualized in figure 4(a). This is a standard validation procedure commonly used in ML practice, and implicitly assumes that all data instances are independent. In this case, the model to be trained is referred as the subject-dependent model [24].

To answer question (b) with an experiment, we would bring the same subject back for another session, such as after a break or on another day. This helps to understand whether the predictions are consistent across sessions. This procedure can again be mimicked in a validation procedure by ensuring that all data from a given session stay in the same split of data. When performed with cross-validation, this is commonly referred to as *group cross-validation*, where the randomization is over groups of data rather than over individual instances. We visualize how this process would be applied in cross-validation in figure 4(b) compared to the instance-level split in figure 4(a), which use the visualization of sessions from figure 3. In the instance-level validation, each subject and session’s data are in the training or testing set on every fold, and there is no structure to which data is held-out at any given point. Conversely, in the case where we split by session, all data from a complete session is either in the training or validation, where this creates structure on which data instances are in the validation or training set on each fold. Unsurprisingly, this is a more challenging task for a ML algorithm, which we show empirically below, as it requires an algorithm to achieve a greater level of generalization. In this case, the model to be trained is referred as the cross-session model [24].

To answer question (c) with an experiment, we would need to bring in new subjects that we had not included before in the experiment and try the algorithm on them in a prospective manner. In a validation procedure, we can mimic this experiment by using complete subjects for validation and test data rather than individual instances or sessions. This would estimate performance of our ML model on unseen subjects’ data, which more closely mimics the motivating experiment and test in practice. This is again a type of *group cross-validation*, where the randomization is done by subjects. We visualize this procedure in figure 4(c), which provides more structure than grouping over sessions. Subject-level validation is more challenging still as it requires further generalization compared with session-level validation within the same subject. In this case, the model to be trained is referred as the subject-independent or cross-subject model, e.g. in BCI for EEG-based emotion recognition or vigilance estimation [22]. However, this type of subject-based validation approach is appropriate for many questions in neuroscience [7]. For smaller datasets, these careful validation strategies are even more critical, as the limited data increases the risk of overfitting and inflating performance metrics.

Finally, we note that it is impossible to ask question (d) from this data without making significant assumptions, as our data all came from a single center. Evaluating models on another center is often referred to a form of external validity, and is a more challenging level of generalization. Such efforts require explicitly comparing and combining data from multiple medical centers. We include it here to highlight that there are always limitations of the data, and that it is important to address the limits of generalization of any method. We note that there are many scales of generalization that are useful in practice, and that a common goal of translational research is to gradually increase the scale of generalization. For example, in addition to the cases mentioned above, this could include generalizing across genotypes, measurement devices, or species [25], which is beyond the discussion here.

A natural follow-up question is how much these differences in validation matter in practice. Like most of statistics, the answer is that *it depends* on the data properties and question; for example, in the case studies below, we show that because electrophysiological signal can as electrophysiological signals may contain information about person-specific traits or conditions [26–28], targets that are constant for a subject across instances (e.g. features allowing diagnosis of mental disorders) require careful adjustment to account for the data structure. Roy *et al* [2] highlighted similar discrepancies in EEG-based DL studies, noting that validation strategies can report highly discrepant values for intra-subject and inter-subject validation procedures. Similarly, the HAMLET framework [29] explored intra-subject and inter-subject validation settings, showing a huge discrepancy in performance between these two cases and demonstrating the challenges of generalizing across subjects. Thus, different validation practices can make a huge difference in practice in our performance estimates under common conditions, often rendering unrealistically optimistic performance estimates.

Focusing on validation practices is essential to explicitly demonstrate how methodological choices impact reported results. Below, we directly examine how validation strategies on several real-world datasets relate to research questions and interact with data structures. By showcasing the exact impact of different cross-validation procedures on performance estimates, we hope to provide researchers with practical insights to design more rigorous and relevant validation strategies.

#### 2.2.1. Animal model of stress response

First, we will look at a task related to brain state, which relates local field potentials collected in mice from implanted electrodes in 11 different brain regions to stress and genotype. This dataset is publicly available [30, 31]. This data was previously used to help design a neurostimulation protocol to mitigate the impact of stress, as described in full elsewhere [32]. Briefly, the data consists of 14 wild-type mice and 12 Clock-$\Delta$19 mice. The Clock-$\Delta$19 genotype is a mouse model of bipolar disorder [33]. Each mouse has a 300 s recording in their home cage, a 300 s recording in an open field test, and 600 s during a tail suspension test. The tail suspension test is used to assess response to a challenging experience [34]. We would like to discover the brain signature relating to stressful conditions and to genotype. We therefore set up the ML problem where $x_{mi}$ represents the derived features for the $i \mathrm{th}$ second of the $m \mathrm{th}$ mouse. Here, there are $M = 26$ subjects and total instances and data was collected in a single session for each subject. These features represent the frequency-based power and frequency-based coherence between brain regions, resulting in 3696 different features [31]. We then define the corresponding target $y_{mi}$ as either the stress condition (open field and tail suspension are considered stressed and home cage as non-stressed) or genotype (wild-type versus Clock-$\Delta$19).

For our purposes here, we choose a standard multi-layer perceptron with a single hidden layer of 10 hidden units with a rectified linear unit (‘ReLU’) nonlinearity, as neural networks are very standardly used models in the literature. We are not performing model selection (e.g. only using one configuration) but rather focusing on the differences in performance estimates due to the validation procedure. It is unlikely that this is an optimal modeling approach; however, our focus here is on the impact of the validation on a reasonable ML procedure rather than finding the optimal predictive model.

To understand the impact of the validation procedure and the importance of carefully choosing the validation procedure, we explore the impact on performance estimates from several different validation procedures where we predict both stress and genotype. First, we show the result from not doing validation, i.e. evaluating the classifier on the training data. Not doing validation results in inappropriate results, and we only include it here as an example of a bad practice that has been responsible for previously retracted papers [35]. Second, we perform a 5-fold cross-validation over instances, which is the strategy implied by question (a) above, i.e. classifying unseen data from the same subject. Third, we use the 5-fold group cross-validation approach over mouse identity, which is the strategy implied by question (c) above, i.e. classifying unseen data from unseen subjects.

We report performance on these binary tasks by using area under the receiver operating characteristic curve (AUROCC, usually simplified to AUC). AUC is a standard ML metric where 1.000 is a perfect classifier, and 0.500 represents classification no better than chance (e.g. no information). The results are shown in table 1. Succinctly, not performing cross-validation results reports near-perfect performance, but it is dramatically incorrect. It may be surprising that the result from not doing validation is essentially perfect, but neural networks routinely overfit to this extent on training data despite reasonable generalization [36].

**Table 1.** Results on multiple prediction tasks on the LFP dataset using a multi-layer perceptron. Parentheses denote standard deviation of the cross-validation splits. The table reports predictive performance under different approaches, including reporting on training data, a 5-fold cross-validation over instances, and 5-fold cross validation over subjects.

Target/metric	Stress/AUC	Genotype/AUC	Subject ID/Acc.	Random/AUC
No validation	1.000 (N/A)	1.000 (N/A)	0.998 (N/A)	1.000 (N/A)
5-fold over instances	0.932 (0.002)	0.980 (0.001)	0.949 (0.001)	0.979 (0.005)
5-fold over subjects	0.886 (0.014)	0.639 (0.118)	N/A	0.579 (0.129)

Next, we note that the performance from the instance-based validation still performs very well, but there is a significant discrepancy between the by-instance *K*-fold and by-subject *K*-fold for both binary tasks. This discrepancy is practically important as the by-subject validation strategy matches the relevant scientific question here, which is how well we could identify a stress condition in a new subject. In the stress prediction case, this discrepancy would imply roughly twice as many errors as predicted by the instance-based validation if the method was applied to a new subject, which is more realistic. Furthermore, the gap between the performance in predicting genotype reported by these two different validation approaches is gigantic. This neural network looks almost perfect in the instance-level validation, whereas the by-subject validation is barely greater than 0.500 with significant uncertainty. We use the standard deviation as a simple measure to convey uncertainty as constructing confidence intervals on predictions from cross-validation data is complex due to the dependence between the folds [37].

This result may at first seem surprising, but it highlights the limitations of the collected data. While the number of instances ($N = 58\,135)$ seems like it is ‘big data’, we are statistically limited by the total number of subjects ($M = 26)$. This is compounded by the uniqueness of brain signatures. In fact, brains are considered as a potential biometric because of their unique signals and responses [26–28]. To demonstrate this effect, we next evaluated whether this same neural network architecture could be adapted to identify the subject from instances of electrophysiological data. This requires adapting the neural network to predict one of many classes rather than a binary through a small coding change. We found that without a validation, it seems again essentially perfect. When using instance-level validation, we found that it could identify the source subject out of the 26 possibilities most of the time (95%), meaning that subject identity is embedded in the neural data. We do not provide group *K*-fold results here, as the performance of this model architecture to predict new subjects is very poor.

**Figure 5.**
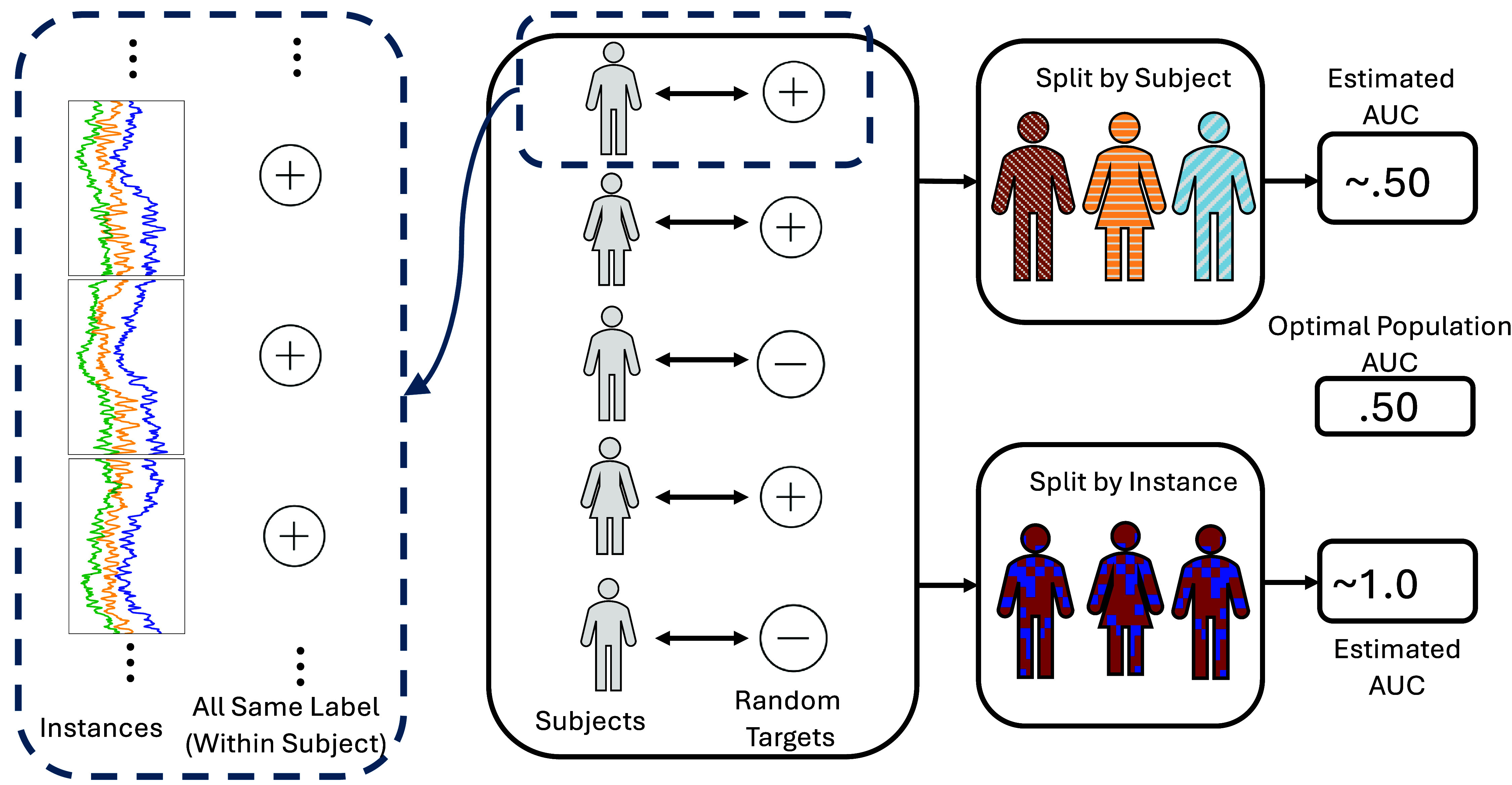
Visualization of subject-level randomization procedure. (middle) Each subject is assigned a random label or outcome, either by shuffling subject-level labels (permutation testing) or by drawing the outcome from a random number generator. (left) In this procedure, each instance of data in a subject is assigned the same label. (right) Using different cross validation procedures results in drastically different performance. Because of the randomization procedure, the optimal population-level accuracy is 50% assuming balanced classes. Splitting by instance yields near perfect predictions as the machine learning system learns to identify individuals rather than identifying information related to the outcome of interest. Splitting by subject yields performance estimates indistinguishable from chance (50%). See table 1 for numerical results on an example case.

This leads to an important conclusion: when predicting subject-level data, the instance-level validation strategy can lead to the classifier just memorizing the subject’s identity, which can confound interpreting subject-level performance. To emphasize this point, we next randomly assigned each subject a binary (artificial and random) label and evaluated how well the neural network could predict these arbitrary labels. This procedure is visualized in figure 5. Here we show that each subject is assigned a random binary target, and each instance in that subject gets the exact same target. As expected, without using a validation set, the AUC implies perfection. Performing validation over instances, though, is misleading, as it reports a stunningly high AUC of 0.979, which is essentially perfect. The neural network accomplishes this task by simply memorizing which subject is which, as the labels have a completely random relationship with the neural data. When validating by subject, the performance estimate clearly overlaps with a 0.500 AUC, so there is no significant evidence that the neural network can truly predict this relationship. It may be surprising that the by-instance validation did even better with a random label than it did predicting subject identity; however, it is worth noting that to predict a random label, you only need to identify a group of subjects rather than an individual, which is an easier task. Second, the fact that the by subject validation was above 0.500 for random targets may be surprising, but this is simply due to random chance with ‘small’ data. With enough independent repeats of this experiment, we would expect that the hold out AUC over subjects would be exactly 0.500.

#### 2.2.2. Emotion recognition

In this section, we explore the task of emotion classification using EEG signals. This analysis involves EEG data recorded from 16 healthy participants who watch video clips rich in emotional content across three sessions. The data used for this task is the SEED-V dataset, as detailed in previous works [24, 38, 39]. During the experiments, participants watch 45 film clips, confirmed through prior studies to consistently evoke specific emotions—happiness, sadness, disgust, neutrality, and fear. The dataset is partitioned into three sessions, each containing 15 clips (trials) to ensure an even distribution of three clips per emotion, thus maintaining a balanced representation of emotional stimuli. We follow data preprocessing and feature extraction as detailed in previous work [38]. Succinctly, the raw EEG data are initially downsampled to a 200 Hz sampling rate. This is followed by the application of a bandpass filter ranging from 1 Hz to 75 Hz to reduce noise and artifacts from the EEG data.

For feature extraction, we use the differential entropy (DE) features from the EEG signals across five frequency bands—delta (1–4 Hz), theta (4–8 Hz), alpha (8–14 Hz), beta (14–31 Hz), and gamma (31–50 Hz)—across all 62 channels, resulting in extracted features of 310 dimensions (62 channels × 5 frequency bands). The ML framework is therefore set up with each feature matrix for each trial, session, and subject, and each of which has its own target emotional class. Here, there are $M = 16$ subjects, 3 sessions and 15 trials per subject, and an instance is considered every 4 s of non-overlapping brain data. This results in $N = 29\,168$ total instances. For modeling, we choose a standard multi-layer perceptron with a single hidden layer of 256 hidden units with a ‘ReLU’ nonlinearity. As mentioned in section 2.2.1, it is unlikely that this is an optimal modeling approach; again, our focus here is on the impact of the validation procedure rather than finding the optimal predictive model.

To understand the impact of the validation procedure and the importance of carefully choosing the appropriate validation procedure, we explore how different validation procedures affect performance estimates in predicting emotion class. First, we show the result from not doing validation. Again, not doing validation results in inappropriate results. Second, we use a *K*-fold cross-validation over instances, which is the strategy implied by question (a) above. This method, although common, may not be ideal for our dataset where multiple instances from the same trial correspond to the same emotion class. Such overlap can lead to overfitting as ‘new’ instances may not be truly independent, having potentially shared information with other instances within the same trial. In other words, some instances from one trial may be assigned to the training set, while other (potentially overlapping) instances from the same trial may be assigned to the test, preventing the training and test sets from being independent. We then employ a *K*-fold group cross-validation strategies that consider different aspects of our dataset’s structure: subject identity, trial identity, session identity, and combinations thereof.

For session identity, we use 3-fold cross-validation reflecting the three available sessions, and 5-fold cross-validation for all other identities, representing different and increasing levels of generalization [24, 38, 39]. The results reporting prediction accuracies are shown in table 2. Our results reiterate that not performing cross-validation or using standard *K*-fold cross-validation often yields misleadingly high performance. These methods fail to test the model against true new instances, highlighting the critical importance of selecting appropriate validation strategies for accurate performance evaluation. Next, we observe a significant performance drop when using *K*-fold group cross-validation. Specifically, using *K*-fold group cross-validation by subject identity, we find that accurately identifying an emotion class in a new subject is markedly challenging, with an accuracy of only 0.318. This result contrasts with those obtained using *K*-fold cross-validation by instance, highlighting the importance of selecting the appropriate cross-validation strategy.

**Table 2.** Results on multiple prediction tasks on the SEED-V dataset using a multi-layer perceptron. Parentheses denote standard deviation of the cross-validation splits. To align our results with those of previous studies, we employ a 3-fold cross-validation by subject. Specifically, for each subject, the 15 film clips in each session are divided into three segments: the first 5 clips, the middle 5 clips, and the last 5 clips. Data from the three sessions are then concatenated. Models are trained on permutations of two of the three segments and tested on the remaining third segment to evaluate performance. $K$ = 5 for all experiments except for session identity, which uses $K$ = 3.

Target/metric	Emotion classification/Acc.	Subject ID/Acc.	Session ID/Acc.	Random by subject/Acc.	Random by session/Acc.
No validation	1.000 (N/A)	1.000 (N/A)	1.000 (N/A)	1.000 (N/A)	1.000 (N/A)
*K*-fold	1.000 (0.000)	1.000 (0.000)	1.000 (0.000)	1.000 (0.000)	1.000 (.000)
3-fold by subject	0.719 (0.096)	N/A	N/A	N/A	N/A
Subject/group *K*-fold	0.318 (0.048)	N/A	0.397 (0.050)	0.209 (0.156)	0.384 (0.084)
Session/group *K*-fold	0.481 (0.029)	0.972 (0.011)	N/A	0.937 (0.027)	N/A
Trial/group *K*-fold	0.494 (0.089)	0.997 (0.004)	0.997 (0.004)	0.996 (0.006)	0.997 (0.004)

As highlighted in section 2.2.1, despite having large number of instances ($N$ = 29 168), our statistical power is significantly constrained by the number of subjects ($M$ = 16). This is compounded by the uniqueness of EEG signals [40, 41]. To demonstrate this effect, we test whether the same neural network architecture could identify subject, trial, and session identities using the provided DE features from EEG signals. We observed that without any form of validation or using standard *K*-fold cross-validation, the model appears to perform perfectly. Interestingly, when using group *K*-fold cross-validation by session or trial, the model is still able to accurately identify the source subject and session identity nearly perfectly and above chance, respectively, indicating that these identities are inherently encoded in the EEG data. This finding further supports the conclusion that instance-level and some group-level validation strategies may memorize subject and session identities rather than learning to generalize.

To further demonstrate the robustness of the different cross-validation procedures, we randomly assign one of the five targets to the data and assess the neural network’s ability to predict random targets when they are structured either by subject, where all instances of a subject’s data are randomly assigned the same target, or by session, where all instances of a session are assigned the same target. The first case is aligned with the prior conclusions above, where only cross-validation at the subject level captures the fact that it is a random relationship. Notably, even splitting by session gave an accuracy of 93.7%, whereas splitting by subject has an accuracy of 20.9% with high uncertainty (compared to a theoretical expectation of 20%). When randomly assigning targets by session, we see trends suggesting that we are not necessarily capturing the relevant information when session is related to target structure. Here, the network can estimate the correct target 38.4% of the time in cross-subject validation, whereas it could estimate the correct session 39.7% of the time. In other words, the network is capturing the structure related to session, not target. This further emphasizes that we need to consider structure of the targets and the research goals when choosing the validation procedure.

#### 2.2.3. Sleep staging

In this section, we explore the task of sleep stage classification using EEG signals to explore the properties that occur when the targets have natural, rather than enforced, structure through time. We classify 30 second segments of polysomnography (PSG), referred to as ‘epochs’, into different sleep stages using the Sleep Cassette (SC) subset of the 2018 Sleep-EDF dataset, also known as SC-EDF-20 [42]. This dataset includes scalp EEG recordings from two channels (Fpz-Cz and Pz-Cz) across 20 healthy participants. Following established protocols [43–49], we consider only the PSG data from 30 min before to 30 min after the recorded sleep period, as the dataset includes a lot of wake data outside this window. The epochs are scored using the Rechtschaffen and Kales rules, with adaptations to align with the American Academy of Sleep Medicine standards by merging stages N3 and N4 into a single N3 stage. We exclude epochs labeled as MOVEMENT or UNKNOWN. We adhere to the preprocessing and feature extraction methods detailed in prior research [43], resulting in features of 1081 dimensions derived from both time and frequency domains, across multiple window sizes. Each feature vector, represents the derived features for the $i \mathrm{th}$ recording of the $m \mathrm{th}$ subject. Here, there are $M = 20$ subjects and $N = 42\,230$ total instances. We then define the corresponding target $y_{mi}$ as the sleep stage, categorized as wakefulness (W), stage N1, stage N2, stage N3, and rapid eye movement (REM). For the model, we use a standard multi-layer perceptron with two hidden layers containing 256 and 10 units respectively, each using a ReLU nonlinearity. Again, it is unlikely that this is an optimal modeling approach; our focus is on evaluating the validation procedure’s impact rather than on identifying the most effective predictive model.

**Figure 6.**
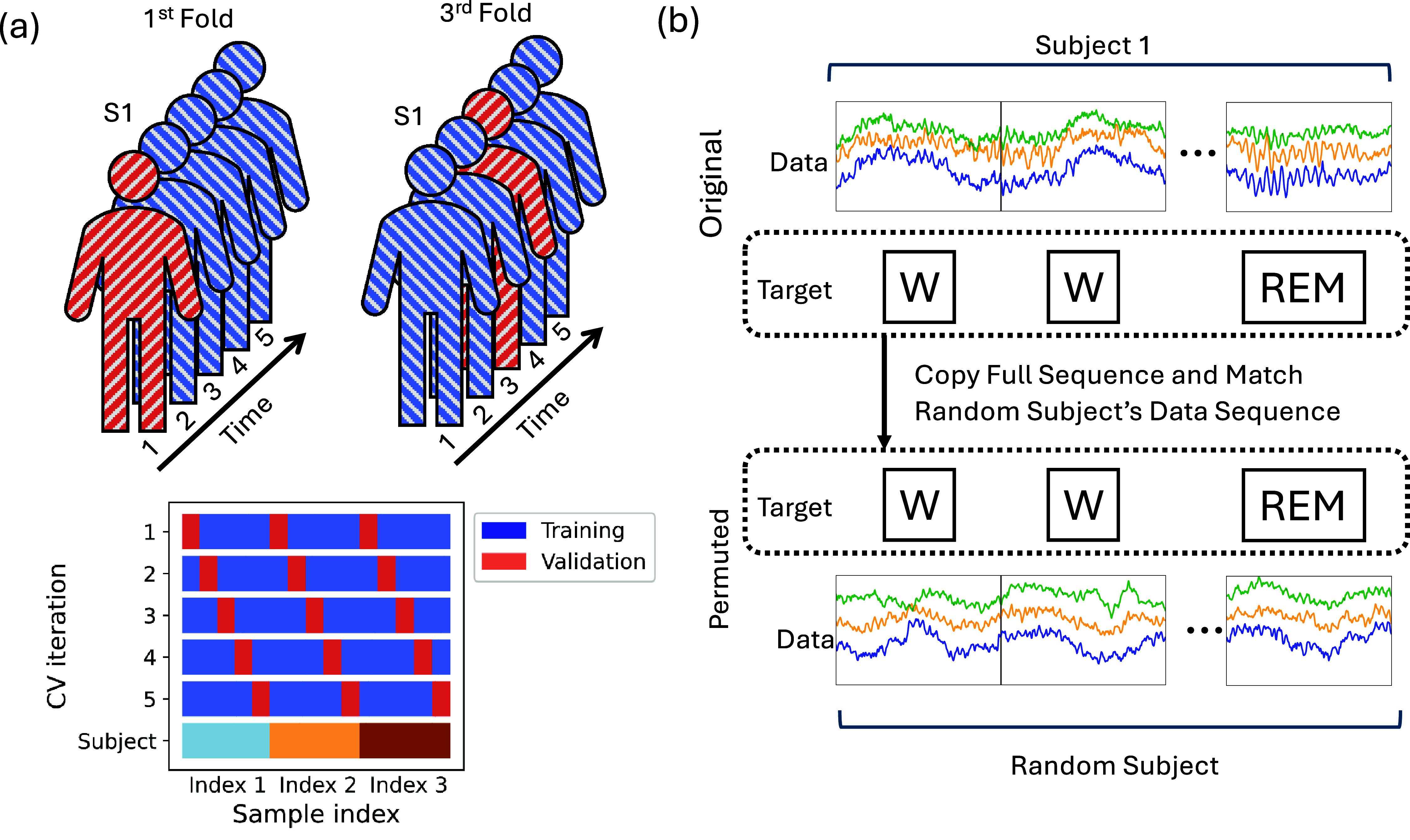
Visualization of time structure and cross-subject permutations. (a) Visualization of a validation split over time, where each subject is split into 5 sequential chunks even though data can be thought of coming from a single session. The top shows subject 1 (S1)’s data in the first fold and third fold of the cross-validation process, showing the evolution of the test set. (b) Visualization of the random permutation between subjects. The top shows a hypothetical relationship between data and the target sleep state (here Wake (W) and rapid eye movement (REM)) as measured. The complete sequence of targets is copied and matched with data from a random subject, which breaks the relationship between the data sequence and the sleep state but keeps the temporal structure.

In this section we evaluate the impact of different validation procedures on the accuracy of sleep stage prediction. Firstly, we reiterate that neglecting to use any validation can lead to unreliable results. Secondly, we use a *K*-fold cross-validation over each instance, as outlined by question (a). Thirdly, we adopt a *K*-fold group cross-validation by subject identity in accordance with question (c). Beyond these methods, we also evaluate how much the temporal patterns in sleep labels impact predictions by doing a time *K*-fold cross-validation, illustrated in figure 6(a). Validating by time is conceptually similar to by-session with the distinction that it is not necessarily following a session structure, but perhaps a single long session. It is commonly used in practice in evaluating sleep [50]. In this approach, instances for each subject are partitioned into $K$ contiguous segments. The models are then trained on four segments and tested on the fifth to measure performance, simulating the chronological sequence found in real-life data streams. This method not only addresses the strategy of question (a) but also investigates the significance of instance order in validation procedure selection. The results reporting prediction accuracies are shown in table 3. Consistent with our earlier findings in sections 2.2.1 and 2.2.2, not performing cross-validation results in perfect performance, but it is misleading due to the inherent properties of neural networks.

**Table 3.** Results on multiple prediction tasks on the SC-EDF-20 dataset using a multi-layer perceptron. Parentheses denote standard deviation of the cross-validation splits. *K* = 5 in all experiments.

Target/metric	Sleep stage/Acc.	Subject ID/Acc.	Subject-random/Acc.	Subject-permutation/Acc.
No validation	1.000 (N/A)	1.000 (N/A)	1.000 (N/A)	1.000 (N/A)
*K*-fold	0.891 (0.003)	0.966 (.0120)	0.969 (0.004)	0.588 (0.017)
Subject/group *K*-fold	0.827 (0.029)	N/A	0.141 (0.096)	0.323 (0.044)
Time *K*-fold	0.831 (0.013)	0.772 (0.060)	0.838 (0.062)	0.383 (0.012)

Next, we observe that instance-based validation performs well; however, a notable performance gap exists between instance-based *K*-fold (Acc: 0.891) and the subject-based group *K*-fold (Acc: 0.827) as well as time *K*-fold (Acc: 0.831) approaches. These gaps are significant since the objective is to accurately identify sleep stages in new subjects, as well as to maintain accuracy within the same subject across a chronological data sequence that mimics real-life situations. To rigorously assess the difference between instance-level and subject-level validation, we applied the corrected resampled *t*-test [51], together with confidence intervals. This method accounts for the dependence between cross-validation folds, which should be considered when dealing with repeated measures in neural data. Here we perform a single round of 5-fold cross-validation (*k*= 5) and estimate the correlation between folds as 1/*k*. This approximation serves as a straightforward way to consider the inherent dependencies in cross-validation arising from shared training data.

Our analysis shows that instance-level *K*-fold cross-validation, while achieving a higher average accuracy (0.891 ± 0.0031), is overly optimistic because it allows the model to learn subject-specific characteristics from the training data. In contrast, subject-level *K*-fold cross-validation requires the model to generalize to unseen subjects, resulting in a lower mean accuracy (0.827 ± 0.029) that more accurately reflects the challenge of generalizing across different individuals. The corrected resampled *t*-test shows a statistically significant difference between these two approaches, with a mean performance difference of 0.064 [95% CI: 0.013, 0.115], with the instance-level approach yielding significantly higher performance estimates (*p* = 0.025). The confidence interval is based on the mean difference between the two approaches and its corrected variance for fold dependencies.

Again, our model is hindered by having only 20 subjects (*M* = 20), especially considering the heterogeneity of EEG signals across individuals [40, 41]. To further investigate this within the SC-EDF-20 dataset, we next evaluate whether this same neural network architecture could be adapted to identify the subject that provided the instance of sleep EEG data. Again, we find that without validation, it is almost perfect. Instance-level validation also demonstrates near-perfect performance in identifying the source subject, verifying that subject-specific characteristics are discernible in the EEG data. However, a notable decrease in accuracy is observed with time *K*-fold cross-validation (Acc: 0.7716), suggesting that while the EEG data contains subject-specific information, the chronological structure of the data also influences model performance. As section 2.2.1, we do not perform group *K*-fold across subject, as it is impossible in such a setting to predict new subjects.

This leads to an important conclusion: when predicting subject-level data, the instance-level validation strategy can lead to the classifier just memorizing the subject’s identity. Following the approach in sections 2.2.1 and 2.2.2, we assign a single random sleep stage labels to each subject and assess the neural network’s predictive accuracy, meaning that each subject is assigned a single random, artificial label. Unsurprisingly, the model implies perfection on a validation set. Based on our enforced random label-data relationship, we would expect our model to generate predictions close to random. However, the high accuracy (0.969) is deceptive for instance-level validation, as it results from the model remembering subject identity rather than learning from the label-data relationship. Subject-based validation yields low accuracy (14.1%), indicating there is no significant evidence that this neural network could truly predict this relationship. Interestingly, instance-level and time *K*-fold cross-validation performed better with random labels than in identifying subject identity. As discussed earlier, this may be because these methods only need to identify a group of subjects rather than an individual, which is inherently less complex.

Next, we assess whether a model is learning meaningful patterns from the data that generalize across individuals, or if it is simply memorizing the data-label pairing for each subject by memorizing points in time. To evaluate this, we apply a label shuffling procedure among the subjects within our dataset, as shown in figure 6(b). For example, we randomly reassign subject $k$’s labels to subject $j$’s data, thereby preserving the temporal structure of the data but deliberately disrupting the inherent link between the data and its corresponding target. Due to the variation in the number of instances per individual, we restrict our analysis to subjects with over 2000 instances. Despite the shuffling, instance-level validation still has 58.8% accuracy over 5 classes, meaning that our model captures noisy temporal patterns that do not depend on the actual label relationships. These results emphasize how critical it is to match the scientific question to the splitting procedure.

#### 2.2.4. Considerations for dataset size

The validation strategies discussed in this section are influenced by the size of the dataset, as different data sizes require adjustments to ensure robust and reliable results. This is especially apparent in the case studies presented earlier, where we are statistically limited by the number of subjects, which varied from 16 to 26. The classification performance gap we observed between splitting strategies in these case studies is designed to convey how impactful careful selection of the scientific question and subsequent cross-validation procedure are on statistical properties. Techniques such as nested cross-validation (see section 2.3) can be particularly useful in these cases.

For larger datasets, the performance gap between different validation strategies is expected to decrease but remains a critical consideration. For instance, in a recent study identifying an EEG biomarker for subjects diagnosed with autism compared to neurotypical controls, we analyzed a dataset with 293 total subjects [52]. Using subject-level cross-validation, the model achieved an AUC of 0.72. However, when evaluated instead using instance-level validation paired with an expressive DL method, the AUC exceeded 0.9. This discrepancy highlights the importance of specifying the correct validation procedure, as improper validation can lead to overly optimistic performance estimates (here the AUC of 0.9), that would not reflect real performances on unseen subjects.

For large datasets, careful consideration of computational demands is crucial, as different validation techniques can significantly impact overall costs. Efficient sampling methods may be necessary to reduce computational burdens while preserving the integrity of the evaluation. In DL, for example, it is common practice to use a single validation split rather than cross-validation to balance these trade-offs. By explicitly addressing such considerations, we can ensure that validation strategies remain both scalable and applicable across diverse neural engineering research contexts.

#### 2.2.5. Considerations for temporal structure

Addressing the inherent temporal structure within neural data needs careful consideration at both the data preprocessing and model selection stages. When partitioning data for validation purposes, it is important to use methods that consider temporal dependencies. One effective method involves partitioning the data into contiguous temporal blocks, ensuring that data points within a defined block stay in the same cross-validation fold. This approach, analogous to the validation strategy previously discussed in the context of sleep stage classification, is essential to prevent data leakage between training and testing sets, which can artificially inflate performance metrics. Furthermore, data organization can be structured around temporal components, such as grouping data within sessions or subjects. This systematic grouping ensures that samples exhibiting similar temporal dependencies are collectively managed, maintaining the integrity of the temporal relationships inherent in the data.

In addition to data splitting considerations, the selection of appropriate modeling techniques is important for effectively capturing temporal dynamics, including many different statistical and DL models. These models possess the capacity to learn from the temporal context embedded within neural signals, thereby enabling the capture of both short-range and long-range temporal dependencies. Such approaches can make a large impact on predictive performance and scientific conclusions.

### 2.3. Challenges in model evaluation

The previous section dealt primarily with the challenges in interpretation due to a variety of ways of data splitting within cross-validation. As can be seen, estimates of performance can highly vary due to the chosen procedure, and it is important to link the scientific question to the procedure for performance to hold up in practice, and for the ML classifier to learn and use task-relevant patterns, and not confounding patterns, e.g. patterns related to subject identity. Note that the latter issue was recently noted in a survey that found that many clinical neural engineering papers using EEG and DL for medical diagnosis were incorrectly splitting their training and testing data [15]. This makes it rather likely that these classifiers were (incorrectly) using the subjects’ identities for classification rather than disease-related EEG features, thus vastly overestimating the diagnosis accuracy.

While we report predictive performance by averaging over the different validation folds in the cross-validation procedure, there are underlying biases to address in this procedure. A downside of using such a cross-validation procedure to report performance is that it will overreport performance when used to select from many different models (e.g. if you compare many models, one of them may look good by random chance) [20]. In fact, despite the common knowledge that ML models improve with more data [53], the reported performance in the literature can decrease with increasing as the bias from the cross-validation procedure decreases [54]. In order words, even though the model is getting better, it could appear worse because the upward performance bias from model selection during the cross-validation decreases.

Historically, a variety of methods have been used to try to improve validation efforts. For example, nested cross-validation [20] has been commonly used to mitigate performance reporting bias and estimating confidence intervals [37]. In nested cross-validation, the model selection procedure has two levels of cross-validation. The first level is the same as in typical cross-validation, and the second level is performed within each cross-validation split. Model selection is performed on the second level within each first level split, and then the first level split is used to estimate performance. This technique has been shown to report more accurate performance. Additionally, it is relatively common to run permutation tests [55], which can be used to estimate what type of predictive performance we might expect with random information. We demonstrate this idea above where we swap the labels between subjects in the sleep data and evaluate their performance but used as a statistical testing procedure. This allows us to evaluate whether the underlying structure impacts our predictive ability. Additionally, it is possible to perform statistical analysis based on the distribution that comes out of repeated permutation testing. We emphasize that when doing permutation testing it is critical to choose which structure is kept in the permutation test (e.g. do we permute over instances, sessions, or subjects), as highlighted in the case studies above.

For a test set with little data, which is often the case in neural engineering, the chance level performance metric should be reported and compared to the actual performance obtained. This is analogous to some of our previous experiments, where we use random targets and permutation tests to evaluate chance performance. For classification accuracy, there are analytical estimates of the chance level [56]. For other performance metrics, or for regression problems, chance level performance should also be reported and could be empirically estimated using permutation tests, as described above.

We also emphasize that there should be careful consideration of the metric chosen to evaluate and compare performance. Above, we choose to largely use accuracy and AUC to illustrate the examples as it is a common metric that is easy to understand and show the behaviors of the different cross-validation techniques. However, it is also common to use metrics based on precision-recall, along with a host of metrics appropriate for comparing modeling performance or the utility of additional information [57]. For unbalanced test data, i.e. with a different number of instances in each class, a performance metric that can handle imbalance (e.g. AUC, balanced accuracy, precision-recall curves, F1 score, etc) should be chosen [22]. Classification accuracy does *not* naturally handle class imbalance, and is thus not suitable to estimate performance on unbalanced data. This metric should be chosen in careful consideration with what the scientific goal or question is. These metrics can be adapted for proper statistical testing, which is relatively straightforward on a true held-out set and requires more complicated procedures under cross-validation estimates [20, 37].

### 2.4. Increasing scales of generalization

Different ways of collecting data and splitting cohorts seek generalization at different scales of the data. For example, we can think about generalizing to future data from the same subject, a new subject, a new population, a new medical center, or a different type of device, etc [25, 58]. Assessing these different scales of generalization requires different data collection efforts and processing, and as highlighted above requires different validation procedures. When venturing into new research areas, it is acceptable and often advisable to begin with investigations that have narrower or smaller levels of generalization. This is because pioneering research topics might not have sufficient data or prior knowledge to make broad generalizations immediately. Instead, focusing on specific cases, subpopulations, or conditions can provide valuable insights and set the foundation for future, more expansive studies. For example, neuromotor interfaces are gradually advancing from individualized computational methods to generic interfaces that work across subjects [59]. However, as the field continues to move forward, it is crucial that researchers explicitly communicate the scale of generalization that they are evaluating and operating at in their studies. They should delineate the bounds of their findings and clarify how these findings and the validation procedures apply within those bounds. This ensures that the research results are not misinterpreted or wrongly extrapolated to broader contexts.

Furthermore, linking the chosen scale of generalization to the overarching scientific question helps justify the chosen scope and reinforces the study’s relevance. By being transparent and intentional about the scope of generalization and how it relates to the chosen validation approaches, researchers can build credibility, foster a clear understanding of their work, and pave the way for successive research that expands on these initial findings.

### 2.5. Generalization in DL

In most ML systems besides DL, the balance between overfitting and validation is carefully monitored, with the expectation both that the validation performance is reasonable but also close to the estimated performance on the training data [16]. In other words, we have balanced overfitting well, so the model performs similarly on the training and validation data. This principle is also echoed in many statistical models. However, DL deviates from this norm. In most real-world scenarios, a well-performing DL model has training error that is essentially zero [18]. This fact underscores the importance of focusing on the validation error independently, irrespective of an apparent overfit on training data, and motivates the checks outlined in this manuscript.

One might wonder how DL models, despite having numerous parameters, can fit limited data so well. Remarkably, neural networks can still generalize well even if they achieve flawless predictions on the training set. There are ongoing theoretical studies to understand the learning mechanics of artificial neural networks [60] and how this fact arises. This phenomenon is further elucidated by the concept of ‘double descent’ [61], where a DL model’s generalization can improve as the number of parameters increases. This scenario is in deep contrast to typical intuition, where increasing the parameterization gives rise to more complicated and more overfit models. These nuances reinforce how robust and dependable validation is necessary in neural engineering applications.

### 2.6. Current research in advanced ML techniques

ML and AI is rapidly advancing, as can be readily seen by the clear advances in computer vision and large language models over the last few years. Many of these advanced techniques are being adapted to tasks of interest in neuroscience and neural engineering. Recent techniques can be used to effectively improve ML performance on tasks of interest; while we encourage the use and further development of modern techniques, we emphasize that it is important to consider how these techniques may alter the assumptions of the data analysis and therefore change the scientific question.

As a concrete example, we will highlight unsupervised domain adaptation [62]. This has been applied by several groups on neural engineering tasks [63, 64], with the general goal of improving how well we can predict labels on a new subject or patient that does not have ground truth labels. The key assumption of such techniques is that we have *unlabeled* data available for a new subject, meaning for example that we have access to the brain recording of a new subject during the training process. We further note that in the case where ML systems are previously pretrained and used in transfer learning, we cannot even guarantee that the new user has not been included in the training data in general, and care should be taken in making such assumptions. In this situation, this methodology can adapt and analyze the difference between training subjects and the validation subject and improve test performance. While this task can exist in practice (or models can be retrained), it changes the scientific question from ‘How well does this method work on a new subject?’ to ‘How well does this method work on a new subject where we retrain with access to the brain recording but not targets?’ Further extensions of these ideas can be more directly relevant to the first question, such as zero-shot learning or domain generalization [65], but our key point is that we need to carefully consider how the training and assumptions of the methodology changes the interpretation of the results.

Additionally, other modern techniques to automate the model search (e.g. neural architecture search [66], Bayesian optimization [67]) can help identify the most promising models and decrease human effort but may exacerbate overreporting results if only a validation set is used by increasing the number of comparisons. These challenges can be addressed by the statistical methods in section 2.3. On this topic, it may be worth reminding that the selection of ML models, hyperparameters and features should be made using only training data (possibly using inner cross-validation on the training data), and not according to the final independent test set performance, nor using all the data together. Selecting them according to classification performance on data including the test set may result in vastly overestimating such performance, including reaching high accuracy even on completely random data without class information [68]. For instance, the architecture of a deep neural network should not be selected according to which one reaches the best accuracy on the test data, but rather the validation data. Likewise, when doing feature selection prior to model training, the input features should not be selected according to which ones are the most discriminant on all the data available, but rather only on the training data to properly estimate performance. Both these examples are commonly seen mistakes, included in some published papers [69].

Finally, we note that large-scale pretraining through self-supervised learning, which have been so prevalent in computer vision and natural language processing, are increasingly becoming feasible in many neural engineering tasks for electrophysiology [70, 71] and are already widely used for computer vision behavior tracking [72–74]. These techniques have preliminarily demonstrated the potential to help improve general representation of physiological signals and generalization of ML models in neural engineering tasks [70, 71, 75, 76].

## 3. Considerations besides validation and generalization

When considering a ML model, model selection is typically based strictly on predictive performance. However, there are many other considerations when choosing and deploying a ML model. While detailed discussion is beyond the scope of this manuscript, we will briefly discuss a few common additional considerations and their relevance in neural engineering.

### 3.1. Interpretability and explainability

DL is by default considered a black box, meaning that it is indecipherable how the algorithm decides on the predicted outcome [77, 78]. In neural engineering, the use of black box methods like DL can be problematic when making high stakes decisions about brain-machine interfaces or neuroprosthetics, as clinicians and engineers may not understand how the model arrives at its conclusions [79]. This lack of explainability can pose risks, especially if the model behaves unexpectedly during real-time neural signal processing or interventions (e.g. suppose data is input incorrectly from a system, but that error cannot be identified because it is a black box). Furthermore, emerging regulation of AI-based systems impose requirements of transparency and explainability in certain applications, including the medical domain. Hence translational research efforts in neural engineering should carefully consider the use of transparent and explainable approaches.

In high-stakes decisions, it is common to focus on trying to open the black box and fully understand how the ML method is working. This is commonly done by considering interpretable methods or explainable AI (XAI) [80]. Interpretability and explainability are sometimes referred to analogously but refer to two distinct concepts. Interpretability describes the degree to which a human can understand the procedure an algorithm uses to generate a result. A decision tree is a clear example of an interpretable model, where each individual step of the model can be understood and transparently communicated. Many other interpretable or ‘glass-box’ models exist [81] and have seen use in high-stakes applications such as high-profile examples in seizure prediction [82]. It is worth noting, however, that these tools may still require significant effort and training to properly interpret [83]. Furthermore, how the features are defined is a key consideration, as model results will be in terms of those features. The design of features in a way that carefully considers how preprocessing impacts scientific interpretation is an active area of research [84]. For interpretation, is it often ideal to use well-validated features, such as common clinical features [82].

Explainability, in contrast, usually focuses on post-hoc explanations of complex models. For example, we can visualize feature importance in a random forest or highlight sections of an image used in a convolutional neural network. Explainability methods have found significant uses in neural engineering applications. For example, we can explain how changes in neural signals relate to depression [85], or develop brain networks of depression [86] or social appetitive behavior [87] that can be used to design neurostimulation protocols. However, such approaches can be inappropriate for high-stakes decisions, as the explanations are not always faithful to the actual mechanisms used to make decisions [78].

Validation strategies can further influence interpretability and explainability. For instance, in subject-level validation, feature importance scores reflect patterns generalizable across subjects, making the results more robust for clinical interpretation. Conversely, in instance-level validation, feature importance may emphasize idiosyncratic subject-specific traits, leading to explanations that are less generalizable to new subjects or contexts. Both approaches can be appropriate depending on the research question at hand.

These validation strategies are also critical for uncovering biotypes [88] or patterns shared by a subset of subjects. For example, subject-level validation can identify features that are consistent across specific subgroups within the data, helping to define shared mechanisms or characteristics that underlie a particular condition. By contrast, instance-level validation may still provide insights into personalized patterns and potential responses to interventions, which can complement subgroup analysis.

XAI can also be a useful tool to assess whether a DL model is biased by confounding factors. For instance, in a motor imagery BCI experiment, Trocellier *et al* [89] showed that including EEG data immediately following a visual instruction cue as training input features led to a biased DL classifier. Indeed, such a classifier would use brain responses to that visual cue, rather than motor cortical activity, which also leads to inflated classification performance, and to an unusable classifier in practice as such an instruction cue is absent in real BCI use. This was discovered using XAI tools, which revealed that this DL model mostly used EEG signals immediately following the cue, in the brain occipital cortex. Removing EEG signals following the cue from the DL input, led to a network with a lower accuracy but using as features EEG signals during the motor imagery task, in the motor areas of the brain. Altogether, this also stresses the need to carefully control for possible confounding factors, e.g. task-related stimulus, when using black-box ML models in neural engineering, as well as to use XAI with DL to confirm the absence of such confounds.

### 3.2. Computational constraints

While large scale ML is well-known to be data hungry, these models also demand significant computational resources [90]. In many scenarios, trading in a minor performance gain in favor of an algorithm that is more computationally efficient improves usability. For instance, BCIs used for motor rehabilitation or communication for paralyzed individuals require rapid and efficient processing to provide real-time feedback to the user. Similarly, neuroprosthetics, which assist patients in regaining sensory or motor functions, depend on swift and efficient algorithms to mimic natural neural responses. Another example can be found in wearable EEG systems used for seizure detection; these devices must operate efficiently to provide timely alerts without draining the battery. Likewise, closed-loop neurostimulation based on current brain patterns is dependent on minimal lag [91]. As the neural engineering field continues to evolve and embrace more portable, wearable, and implantable devices, it is necessary to ensure computational efficiency of ML algorithms. This is especially important in the context of whether an algorithm is feasibly an online or real-time algorithm, a crucial consideration in many applications.

In addition to processing time, for classifying new data, the time and computational resources needed to train the ML models should also be considered. Notably, given the large variability of neural signals, both across and within users, many ML models in neural engineering are currently subject-specific and/or require frequent re-training to track such variabilities [92, 93]. For those models, the time needed to (re)train the model should be as short as possible, typically a couple minutes maximum, as during such (re)training, the user is waiting to actually use the system. Therefore, algorithms that requires hours or days to be trained would not be suitable in such real-life use situations.

The chosen validation procedure significantly impacts training time, particularly in cross-validation compared to a single validation split. For instance, in $k$-fold cross-validation—where $k$ is the number of folds and $N$ is the total number of data points—the computational complexity scales linearly with both $k$ and $N$. However, $k$ has a more pronounced effect, as the model must be trained $k$ distinct times, and the number of folds is a parameter directly selectable by the researcher. Doubling the number of folds effectively doubles the total computation time. This scaling applies to both subject-level and instance-level splits, though the per-fold training cost may vary depending on the split type.

Additionally, the complexity of the chosen ML method greatly influences computational requirements. Neural networks, for example, have training costs determined by factors such as the number of parameters, the depth of the architecture, and the number of training epochs. More complex architectures, such as transformers, typically incur higher per-sample training costs than simpler models. Therefore, in neural engineering applications, selecting an algorithm that balances model complexity, training time, and computational efficiency is critical to achieving real-time responsiveness and overall usability.

### 3.3. Fairness

ML models, by their very nature, are heavily influenced by the data on which they are trained. Language models, for instance, inherit biases from their human-created training data, leading to significant and sometimes problematic biases in real-world applications [94]. While neural engineering might seem removed from such human-centric biases, data cohorts can still be skewed and non-representative. Academic research experiments are often conducted on cohorts of volunteer students that may not represent the characteristics of the target population in terms of their age, gender, ethnicity, among others. Data quality may not be uniformly high-quality across all groups of individuals. For instance, EEG devices have been observed to produce different results based on varying factors like hair type and scalp thickness [95], while near-infrared spectroscopy has been shown to often provide lower data quality on subjects with darker skin [96].

In medicine more broadly, it is becoming common to run a series of checks for potential ML model bias [97]. These checks can likewise be applied in neural engineering applications when the total number of subjects is high enough to reliably estimate such differences. Recognizing these potential biases early allows researchers to address them proactively, either by refining the algorithms, recommending more balanced data collection, or implementing other bias-mitigation strategies. One approach to this challenge is to identify appropriate fairness metrics and evaluating them on validation and test data [98]. By taking such proactive steps, we can ensure that the results and conclusions derived from neural engineering applications are as fair and representative as possible.

### 3.4. Privacy

Brain data holds a unique position in the realm of biomedical data due to its intimate connection to personal identity, thoughts, emotions, and potentially even an individual’s predisposition to certain conditions or behaviors [99, 100]. With the growth of ML and its capacity to extract patterns from vast datasets, there is a genuine risk of deciphering personal information that a participant might not have explicitly chosen to share. As such, algorithms designed to process brain data need to include measures for protecting the privacy of individuals, including limiting the use of sensitive data, and privacy-conserving mechanisms [101].

### 3.5. Usability and cost

While many ML methods are used as a step towards scientific discovery or as a preprocessing step, the final goal is often to build an implementable algorithm. The usability of a ML model is multifaceted. While predictive accuracy is often a primary focus, the complexity of implementing the model can influence its practicality. As highlighted above, a seizure risk tool has been adopted both due to its interpretability but also its ease of implementation [82]. A model with state-of-the-art accuracy but immense computational demands might be impractical for real-time applications. Conversely, a simpler, more streamlined model might be easier to deploy and maintain in many applications. Additionally, the ease of integrating the model into existing systems, and the potential need for specialized hardware or software, can influence its usability. Implementation science, which studies the methods to promote the adoption and integration of research findings into healthcare practices, underscores the importance of these considerations, and can provide valuable insights for ML deployment in neural engineering [102]. Balancing the aspects of implementation complexity, computational cost, and model performance is crucial to ensure that ML tools are both effective and practical in the neural engineering landscape.

## 4. Reporting guidelines

### 4.1. Existing ML reporting guidelines

Predictive modeling as a subfield has many guidelines for reporting results, such as the Transparent Reporting of a multivariable prediction model for Individual Prognosis or Diagnosis (TRIPOD) statement [9] and Standards for Reporting of Diagnostic Accuracy Studies (STARD) [10]. These frameworks provide valuable general guidance for reporting in predictive modeling and diagnostic studies. However, they do not address the unique challenges of neural engineering, which often involve small subject numbers, complex data structures, and dependencies between data points.

The ML community has itself developed significant guidance on reporting. An important ML venue, NeurIPS, has developed and previously reported on their reproducibility program [8]. This system was largely based on the ML reproducibility checklist. This checklist requires authors at the time of submission to provide responses to many questions on all models and algorithms, theoretical claims, datasets, whether code to recreate experiments was included, and many details on the experimental results. After NeurIPS 2019, roughly one third of reviewers reported that they found the author checklist helpful, and reviewers gave more positive reviews to papers if they found the answers to the checklist complete and useful [8].

While the ML reproducibility checklist has been instrumental in promoting reproducibility in the broader ML community, it lacks specific guidance on validating scientific conclusions derived from neural data. Initiatives such as the DL-EEG checklist address specific reporting needs for DL applied to EEG data, including algorithmic transparency and reporting consistency, but do not explicitly focus on validation process or ensuring the validity of scientific conclusions.

### 4.2. NERVE-ML checklist: purpose and scope

Our NERVE-ML checklist is designed to fill these gaps, offering targeted guidance to ensure that ML methods are both reproducible and scientifically sound within the context of neural engineering. Here we will focus more narrowly on challenges that arise in ML and AI models for neural engineering. Our goal is twofold: (1) encourage reproducibility and (2) help make sure scientific and statistical conclusions are valid. As has been frequently pointed out in ML, a reproducible result does not ensure that it is correct. For example, including code with a paper can replicate results shown in the manuscript, but this will simply replicate any coding error. Likewise, reproducing results does not guarantee their conclusions are correct, as any flaws in methodology or validation strategy will still exist.

Hence, our checklist is focused on both the reproducibility of the ML methods and the scientific conclusions derived from their results. We present the NERVE-ML checklist, which is shown in table 4.

**Table 4.** NERVE-ML (neural engineering reproducibility and validity essentials for machine learning) checklist.

Question	Yes	No	Unclear	N/A
*On data and definition of training and testing set:*				

Is there appropriate detail on all data sources (e.g. origin of the data, relevant summary statistics, preprocessing, etc)?				
Training and testing data should be statistically independent, such that they are not only different data but also not dependent on each other. In particular:				
For data based on epochs/time windows of neural signals (as typical in neural engineering applications), training epochs and testing epochs do not overlap and are not consecutive to each other.				
If the data acquisition protocol has a block structure (e.g. same class label for all data points of that block), then the test data comes from different blocks than the training data.				

*On parameters and hyperparameters selection and optimization:*				

Are the assumptions of the machine learning approaches or models clearly defined and documented?				
In general, the parameters and settings/hyperparameters should be selected on training/validation data that are completely independent of the testing data. In particular:				
Feature selection was performed using training data only (i.e. not using testing data and not using all data)				
Machine learning settings/hyperparameters are optimized only on training data. This should be the case not only for a classifier, but also for any algorithm whose settings are optimized on data.				
Any normalization parameters (e.g. data mean and standard deviation) was estimated on training data only. Testing data should not be included in this estimation (e.g. this estimation should not be made on all available data)				
Hyperparameters (e.g. regularization parameters of SVM or Logistic regression, learning rate, batch size, etc) were selected on training and/or validation data only.				
The architecture of a neural network was chosen on training/validation data only.				

*On performance metrics:*				

Are the chosen performance metrics clearly defined and motivated?				
For unbalanced test data (e.g. a significantly different amount of testing data per class), was a performance metric that can handle unbalanced data used (e.g. area under the ROC curve or balanced accuracy)?				
For a test set with little data, was the chance level performance metric reported (either through analytical calculations or permutation testing) and compared to the actual performance?				
Are measures of uncertainty clearly defined and appropriate?				

*On scientific conclusions*:				

Are the key scientific questions clearly related to model results?				
Is it clear how the validation technique relates to the scientific questions?				
Is the practical significance of the performance metrics clearly stated?				
Are statistics run on model results?				
Are statistical assumptions clearly defined?				
Is it clear what level of generalization (e.g. inter-subject, inter-cohort, inter-genotype, etc) is tested?				
Are the limitations of the results clearly stated?				
Are alternative explanations for performance considered?				
Are there concerns about bias or fairness?				
Were specific metrics on bias and fairness identified and evaluated?				

*On future use of results and developed technologies:*				

Will the data be publicly available?				
Will data include necessary metadata for reproducing results?				
Has the data been properly anonymized according to data privacy protection laws and good practices?				
Does anonymization of the data prevent reproduction of the results?				
Is the data stored in a trustworthy repository?				
Is the data license stated?				
Will the code be released in full, including training code, evaluation code, and visualizations?				
Is the code license stated?				
Are dependencies and software versions included?				
Is it stated which results can be fully replicated by the code?				
Is there an appropriately detailed guide on how to use the code?				
Will the trained machine learning model be released?				
Is it clear what resources are necessary to run the code (computational resources, software environment, computational complexity)?				
Is it stated how long any released material will be available?				
It is stated whether permanent record will be created, typically a digital object identifier (DOI)?				

The NERVE-ML checklist is designed to ensure that key details related to data handling, ML methodology, and scientific conclusions are effectively conveyed. The checklist is divided into five categories, each addressing critical aspects of ML applications in neural engineering, to promote proper use, validation, and scientific conclusions. While the NERVE-ML checklist provides general guidance, we acknowledge that different neural data modalities, such as EEG, LFP, and fMRI, may require additional considerations. Future efforts could build on this work by developing supplementary materials tailored to these specific challenges.

### 4.3. Checklist categories

#### 4.3.1. On data and definition of training and testing set

The first category, **‘On data and definition of training and testing set**’, focuses on how data is collected, processed, and split for analysis. As discussed in section 2.2, proper data splitting is crucial to ensure that validation strategies align with the scientific question. For example, subject-level splits are necessary for generalization across individuals, while session-level splits are more appropriate for assessing temporal consistency within subjects. This category builds directly on the examples and discussions in section 2.2 by ensuring that these considerations are documented and reported. Proper handling and reporting of these steps are critical in neural engineering, where small datasets, dependencies between samples, and potential biases can significantly affect outcomes. Explicitly addressing these factors helps prevent common errors, such as inflated performance metrics due to improper data splits.

#### 4.3.2. On parameters and hyperparameters selection and optimization

The second category, **‘On parameters and hyperparameters selection and optimization**’, addresses the choices made during model development. While our example cases in section 2.2 do not focus specifically on hyperparameter tuning, they emphasize the importance of validation strategies, which are tightly connected to optimization procedures. This category overlaps with recommendations from prior checklists, such as the ML reproducibility checklist and EEG-DL. While the proposed NERVE-ML checklist takes a broader approach by also focusing on scientific validity, this overlap reinforces the importance of continuing well-established best practices and adapting them to the specific challenges of neural engineering. By specifying whether hyperparameter tuning was conducted on independent validation sets (that are different from the final independent test set) and clearly reporting these procedures, this category ensures transparency and helps avoid overfitting or improperly tuned models, as also discussed in section 2.6.

#### 4.3.3. On performance metrics

The third category, **‘On performance metrics**’, ensures that appropriate and interpretable evaluation metrics are reported, as discussed in section 2.3. As also discussed in section 2.2, validation strategies play a critical role in shaping performance estimates, making it essential to align metrics with the research question. For instance, in clinical applications, metrics like sensitivity and specificity are often necessary to assess real-world utility. This category emphasizes not only the careful selection of relevant metrics but also the importance of reporting confidence intervals or statistical significance, providing a clearer picture of the robustness and reliability of the results. This aligns with broader reporting frameworks like TRIPOD and STARD, which emphasize clear and consistent reporting of performance metrics in predictive modeling and diagnostic studies. This transparency ultimately supports better comparisons across studies and strengthens the reproducibility of ML in neural engineering.

#### 4.3.4. On scientific conclusions

The fourth category, **‘On scientific conclusions**’, emphasizes the critical need to link reported results to the underlying research question, ensuring that conclusions are both valid and appropriately scoped. This category highlights the importance of transparently addressing the assumptions, limitations, and generalizability of the findings, as these factors are essential for drawing scientifically rigorous conclusions in neural engineering. For example, studies employing subject-level validation can reliably infer patterns that generalize across individuals within a dataset but should avoid making claims about external generalizability without additional validation, such as multi-center data or external datasets. Similarly, claims about causal mechanisms or clinical applicability must be firmly grounded in the study design and data constraints.

In neural engineering, where datasets are often small and heterogeneous, overstated or unsupported conclusions can have significant consequences, including misguiding follow-up studies or translational applications. To mitigate this risk, this category encourages authors to carefully document how their validation strategies and data structures influence the scope of their claims. This includes clearly distinguishing between exploratory and confirmatory findings and identifying any potential confounding factors that could bias the results. For instance, if confounding factors such as subject identity are not accounted for, the conclusions drawn about feature importance or model performance may be misleading.

This category also places a strong emphasis on ensuring that conclusions are actionable and meaningful within the context of neural engineering applications. For example, claims about performance improvements or clinical relevance should be supported by metrics that are aligned with the research question and target application. Additionally, researchers are encouraged to articulate how their findings could inform future research, practical applications, or clinical implementation, while clearly acknowledging any limitations or conditions under which the results may not generalize.

The NERVE-ML checklist makes a unique contribution by addressing challenges specific to neural engineering, such as subject heterogeneity, complex data dependencies, and the need to balance reproducibility with scientific validity. While frameworks like TRIPOD and STARD emphasize transparency in linking predictive modeling results to scientific or clinical applications, NERVE-ML extends these principles to account for the nuances of neural engineering datasets and methods. By requiring researchers to explicitly connect their results to the underlying research question and application context, this category ensures conclusions are rigorously supported, transparently presented, and meaningful for advancing the field.

#### 4.3.5. On future use of results and developed technologies

The final category, **‘On future use of results and developed technologies**’, emphasizes the practical and translational implications of the study’s findings. It encourages researchers to specify the intended context of use, such as clinical applications or broader research settings, while being transparent about limitations that could impact generalizability or scalability, including small datasets or dependencies on specific devices. This category also highlights the importance of addressing ethical and societal considerations, such as patient safety, data privacy, and equity of access, to align research with responsible innovation. We note that released data and code should be in trustworthy repositories [103]. While we recognize that some responses in this category may appropriately be ‘no’ due to privacy, proprietary, or other constraints, explicitly addressing these issues ensures clarity and trustworthiness. While there is some overlap with TRIPOD and STARD, this category of NERVE-ML uniquely asks researchers to document potential barriers to translating ML models to clinical or real-world use, such as the compatibility of preprocessing pipelines with other systems or the impact of hardware variability. By focusing on how results and technologies can be meaningfully deployed, this category ensures that findings are not only scientifically valid but also impactful and sustainable in the long term.

While this checklist includes many common items and scenarios, it is both non-exhaustive and non-universal. We acknowledge that not all items will apply to every study, and some may not be answerable due to constraints such as privacy or proprietary data. Rather than being prescriptive, this checklist is intended as a starting point to ensure that common issues are addressed and that inappropriate conclusions are avoided due to improper ML techniques.

Altogether, we hope this checklist will help scientists using ML in neural engineering to design valid and reproducible studies. Additionally, it can serve as a valuable tool for reviewers to identify potential biases and methodological flaws in neural engineering submissions, ultimately reducing the number of flawed publications in the field. By fostering rigorous reporting and validation practices, the NERVE-ML checklist directly supports our overarching goals of ensuring reproducibility and validity in ML research. Furthermore, by addressing the unique challenges of neural engineering, it contributes to advancing the field through transparent, trustworthy, and impactful applications of ML.

Finally, we note that not all the additional considerations highlighted in section 3 are included in this checklist. While those are highly important considerations, they are often specific to the task at hand; hence we choose to focus mainly on the reproducibility and validity for the NERVE-ML checklist. We would encourage researchers to still heavily consider such issues in their work.

## 5. Discussion and conclusion

ML has the potential to significantly advance science, particularly in the field of neural engineering. However, this potential can only be realized if ML is applied with transparency, reproducibility, and scientific validity. Throughout this manuscript, we have highlighted common challenges in ML research, as well as issues specific to neural engineering, such as subject-specific, session-specific, and temporally smooth trends in brain data that may not reflect the outcomes of interest. As we demonstrated, the inappropriate application of validation techniques can lead to drastically inflated performance estimates, undermining the reliability of scientific conclusions.

The NERVE-ML checklist, presented in this paper, offers targeted guidelines to address these challenges by focusing on both reproducibility and validity. It builds on existing frameworks, while addressing the unique characteristics of neural engineering datasets, including small subject numbers, complex dependencies, and heterogeneous data. While initiatives like MOABB [14] focus on benchmarking algorithms for BCI research, NERVE-ML takes a broader perspective by addressing reproducibility and validation across the wider range of neural engineering applications. By emphasizing practical considerations such as validation strategies, performance metrics, and the scope of scientific conclusions, the checklist ensures that ML research contributes meaningfully to the advancement of the field. As a next step, developing modality-specific guidelines or appendices could enhance the NERVE-ML checklist, as modalities like EEG, LFP, and fMRI present unique challenges. Future work could expand the current checklist to offer tailored guidance for these data types, ensuring reproducibility and validity across the myriad applications of ML in neural engineering.

In addition to guiding researchers, the NERVE-ML checklist serves as a valuable resource for reviewers, scientific publishers and funding agencies by providing a structured framework to identify potential methodological flaws and biases in submissions. By fostering rigorous and transparent practices, we hope the checklist will not only reduce the prevalence of flawed research but also contribute to a culture of robust, replicable, and impactful ML applications in neural engineering. Moving forward, we encourage researchers to integrate these guidelines into their work while continuing to consider broader ethical and societal implications to ensure that the field progresses in a responsible and meaningful way.

## Data Availability

No new data were created or analyzed in this study.

## Appendix

**Glossary of key terms**

• **Training set:** A subset of the dataset used to train the model. The training set is utilized to learn the model’s parameters, aiming to minimize errors in predictions during the training process.
• **Validation set:** A subset of data used to provide an unbiased evaluation of a model fit on the training dataset while tuning model hyperparameters. The validation set is used repeatedly to estimate the performance of multiple models or model configurations.
• **Test set:** A subset of data used only once to assess the performance of a fully trained model, providing an unbiased evaluation of the final model’s performance on new, previously unseen data.
• **Cross-validation:** A statistical method used to evaluate performance of machine learning models. It is commonly used when the dataset is limited in size. The data set is divided into $k$ subsets (folds), and the holdout method is repeated *k* times. Each time, one of the $k$ subsets (folds) is used as the test set and the other $k$ − 1 subsets (folds) are put together to form a training set.
• **Generalization error:** The measures of how accurately a machine learning model performs on new, unseen data. It reflects the ability of the model to adapt to the variability in new data beyond the training set.
• **Hyperparameters:** Parameters that define the model architecture and how the model is learned, including learning rate, number of layers, number of nodes in each layer, etc. These parameters are set before training the model.
• **Overfitting:** A modeling error in machine learning which occurs when a function is too closely fitted to a limited set of data points. It usually results in a model that performs well on training data but poorly on unseen data due to its complex structure.
• **Confidence interval:** An interval that is likely to contain the true population parameter (e.g. model performance) with a certain level of confidence. Confidence intervals provide a measure of the uncertainty in performance estimate

## References

[1] Ioannidis J P A (2005). Why most published research findings are false. PLoS Med..

[2] Roy Y, Banville H, Albuquerque I, Gramfort A, Falk T H, Faubert J (2019). Deep learning-based electroencephalography analysis: a systematic review. J. Neural Eng..

[3] Collins F S, Tabak L A (2014). Policy: NIH plans to enhance reproducibility. Nature.

[4] United Nations Educational, S and C O (2022). Recommendation on the ethics of artificial intelligence. https://www.unesco.org/open-.

[5] World Health Organization (2021). Ethics and Governance of Artificial Intelligence for Health WHO Guidance.

[6] Glaser J I, Benjamin A S, Farhoodi R, Kording K P (2019). The roles of supervised machine learning in systems neuroscience. Prog. Neurobiol..

[7] Vu M-A T (2018). A shared vision for machine learning in neuroscience. J. Neurosci..

[8] Pineau J (2021). Improving reproducibility in machine learning research (a report from the NeurIPS 2019 reproducibility program). J. Mach. Learn. Res..

[9] Collins G S, Reitsma J B, Altman D G, Moons K G M (2015). Transparent reporting of a multivariable prediction model for individual prognosis or diagnosis (TRIPOD): the TRIPOD statement. Ann. Intern. Med..

[10] Cohen J F (2016). STARD 2015 guidelines for reporting diagnostic accuracy studies: explanation and elaboration. BMJ Open.

[11] Collins G S (2021). Protocol for development of a reporting guideline (TRIPOD-AI) and risk of bias tool (PROBAST-AI) for diagnostic and prognostic prediction model studies based on artificial intelligence. BMJ Open.

[12] Jayaram V, Barachant A (2018). MOABB: trustworthy algorithm benchmarking for BCIs. J. Neural Eng..

[13] Sylvain C (2024). The largest EEG-based BCI reproducibility study for open science: the MOABB benchmark. HAL: hal-04537061.

[14] Aristimunha B (2023). Mother of all BCI benchmarks (MOABB).

[15] Brookshire G, Kasper J, Blauch N M, Wu Y C, Glatt R, Merrill D A, Gerrol S, Yoder K J, Quirk C, Lucero C (2024). Data leakage in deep learning studies of translational EEG. Front. Neurosci..

[16] Hastie T, Tibshirani R, Friedman J (2009). The Elements of Statistical Learning.

[17] Deng J (2009). ImageNet: a large-scale hierarchical image database.

[18] Zhang C, Bengio S, Hardt M, Recht B, Vinyals O (2021). Understanding deep learning (still) requires rethinking generalization. Commun. ACM.

[19] Varma S, Simon R (2006). Bias in error estimation when using cross-validation for model selection. BMC Bioinf..

[20] Krstajic D, Buturovic L J, Leahy D E, Thomas S (2014). Cross-validation pitfalls when selecting and assessing regression and classification models. J. Cheminform..

[21] Varoquaux G, Raamana P R, Engemann D A, Hoyos-Idrobo A, Schwartz Y, Thirion B (2017). Assessing and tuning brain decoders: cross-validation, caveats, and guidelines. Neuroimage.

[22] Thomas E, Dyson M, Clerc M (2013). An analysis of performance evaluation for motor-imagery based BCI. J. Neural Eng..

[23] Varoquaux G (2018). Cross-validation failure: small sample sizes lead to large error bars. Neuroimage.

[24] Li T-H, Liu W, Zheng W L, Lu B L (2019). Classification of five emotions from EEG and eye movement signals: discrimination ability and stability over time.

[25] Walder-Christensen K (2024). Electome network factors: capturing emotional brain networks related to health and disease. Cell Reports Methods.

[26] Bashar M K, Chiaki I, Yoshida H (2016). Human identification from brain EEG signals using advanced machine learning method EEG-based biometrics.

[27] Palaniappan R, Mandic D P (2007). Biometrics from brain electrical activity: a machine learning approach. IEEE Trans. Pattern Anal. Mach. Intell..

[28] Gui Q, Ruiz-Blondet M V, Laszlo S, Jin Z (2019). A survey on brain biometrics. ACM Comput. Surv..

[29] Deiss O (2018). HAMLET: interpretable human and machine co-learning technique.

[30] Carlson D, Kumar S, Dzirasa K (2023). Multi-region local field potential recordings during a tail-suspension test. Duke Res. Data Repository.

[31] Talbot A, Dunson D, Dzirasa K, Carlson D (2023). Estimating a brain network predictive of stress and genotype with supervised autoencoders. J. R. Stat. Soc. C.

[32] Carlson D (2017). Dynamically timed stimulation of corticolimbic circuitry activates a stress-compensatory pathway. Biol. Psychiatry.

[33] van Enkhuizen J, Minassian A, Young J W (2013). Further evidence for Clock-Δ19 mice as a model for bipolar disorder mania using cross-species tests of exploration and sensorimotor gating. Behav. Brain Res..

[34] Abelaira H M, Reus G Z, Quevedo J (2013). Animal models as tools to study the pathophysiology of depression. Rev. Bras Psiquiatr..

[35] Nakanishi M, Xu M, Wang Y, Chiang K-J, Han J, Jung T-P (2020). Questionable classification accuracy reported in ‘designing a sum of squared correlations framework for enhancing SSVEP-based BCIs’. IEEE Trans. Neural Syst. Rehabil. Eng..

[36] Zhang C, Bengio S, Hardt M, Recht B, Vinyals O (2016). Understanding deep learning requires rethinking generalization.

[37] Bates S, Hastie T, Tibshirani R (2024). Cross-validation: what does it estimate and how well does it do it?. J. Am. Stat. Assoc..

[38] Liu W, Qiu J L, Zheng W L, Lu B L (2022). Comparing recognition performance and robustness of multimodal deep learning models for multimodal emotion recognition. IEEE Trans. Cogn. Dev. Syst..

[39] Zhao L M, Li R, Zheng W L, Lu B L (2019). Classification of five emotions from EEG and eye movement signals: complementary representation properties.

[40] Benomar M, Cao S, Vishwanath M, Vo K, Cao H (2022). Investigation of EEG-based biometric identification using state-of-the-art neural architectures on a real-time raspberry pi-based system. Sensors.

[41] Hema C R, Osman A A (2010). Single trial analysis on EEG signatures to identify individuals.

[42] Kemp B, Zwinderman A H, Tuk B, Kamphuisen H A C, Oberyé J J L (2000). Analysis of a sleep-dependent neuronal feedback loop: the slow-wave microcontinuity of the EEG. IEEE Trans. Biomed. Eng..

[43] Van Der Donckt J, Van Der Donckt J, Deprost E, Vandenbussche N, Rademaker M, Vandewiele G, Van Hoecke S (2023). Do not sleep on traditional machine learning: simple and interpretable techniques are competitive to deep learning for sleep scoring. Biomed. Signal Process. Control.

[44] Supratak A, Dong H, Wu C, Guo Y (2017). DeepSleepNet: a model for automatic sleep stage scoring based on raw single-channel EEG. IEEE Trans. Neural Syst. Rehabil. Eng..

[45] Supratak A, Guo Y (2020). TinySleepNet: an efficient deep learning model for sleep stage scoring based on raw single-channel EEG.

[46] Perslev M, Darkner S, Kempfner L, Nikolic M, Jennum P J, Igel C (2021). U-Sleep: resilient high-frequency sleep staging. npj Digit. Med..

[47] Phan H, Mikkelsen K, Chen O Y, Koch P, Mertins A, De Vos M (2022). SleepTransformer: automatic sleep staging with interpretability and uncertainty quantification. IEEE Trans. Biomed. Eng..

[48] Phan H (2022). XSleepNet: multi-view sequential model for automatic sleep staging. IEEE Trans. Pattern Anal. Mach. Intell..

[49] Guillot A, Thorey V (2021). RobustSleepNet: transfer learning for automated sleep staging at scale. IEEE Trans. Neural Syst. Rehabil. Eng..

[50] Parks D F (2024). A nonoscillatory, millisecond-scale embedding of brain state provides insight into behavior. Nat. Neurosci..

[51] Nadeau C, Bengio Y (1999). Inference for the generalization error. https://proceedings.neurips.cc/paper_files/paper/1999/file/7d12b66d3df6af8d429c1a357d8b9e1a-Paper.pdf.

[52] Carson W E, Major S, Akkineni H, Fung H, Peters E, Carpenter K L H, Dawson G, Carlson D E (2024). Model selection to achieve reproducible associations between resting state EEG features and autism. Sci. Rep..

[53] Halevy A, Norvig P, Pereira F (2009). The unreasonable effectiveness of data. IEEE Intell. Syst..

[54] Vabalas A, Gowen E, Poliakoff E, Casson A J (2019). Machine learning algorithm validation with a limited sample size. PLoS One.

[55] Golland P, Fischl B (2003). Permutation tests for classification: towards statistical significance in image-based studies. Biennial International Conference on Information Processing in Medical Imaging.

[56] Combrisson E, Jerbi K (2015). Exceeding chance level by chance: the caveat of theoretical chance levels in brain signal classification and statistical assessment of decoding accuracy. J. Neurosci. Methods.

[57] Pencina M J, D’Agostino R B, D’Agostino R B, Vasan R S (2008). Evaluating the added predictive ability of a new marker: from area under the ROC curve to reclassification and beyond. Stat. Med..

[58] Siontis G C M, Tzoulaki I, Castaldi P J, Ioannidis J P A (2015). External validation of new risk prediction models is infrequent and reveals worse prognostic discrimination. J. Clin. Epidemiol..

[59] Labs C, Sussillo D, Kaifosh P, Reardon T (2024). A generic noninvasive neuromotor interface for human-computer interaction. bioRxiv.

[60] Jacot A, Gabriel F, Hongler C (2018). Neural tangent kernel: convergence and generalization in neural networks.

[61] Belkin M, Hsu D, Ma S, Mandal S (2019). Reconciling modern machine-learning practice and the classical bias–variance trade-off. Proc. Natl Acad. Sci..

[62] Ganin Y (2016). Domain-adversarial training of neural networks. J. Mach. Learn. Res..

[63] Li Y, Murias M, Major S, Dawson G, Carlson D E (2018). Extracting relationships by multi-domain matching.

[64] Ash J T, Schapire R E, Engelhardt B E (2016). Unsupervised domain adaptation using approximate label matching. https://arxiv.org/abs/1602.04889.

[65] Li D, Yang Y, Song Y-Z, Hospedales T M (2018). Learning to generalize: meta-learning for domain generalization.

[66] Zoph B, Le Google Brain Q V (2017). Neural architecture search with reinforcement learning.

[67] Shahriari B, Swersky K, Wang Z, Adams R P, de Freitas N (2015). Taking the human out of the loop: a review of Bayesian optimization. Proc. IEEE.

[68] Dominguez L G (2009). On the risk of extracting relevant information from random data. J. Neural Eng..

[69] Chau T, Damouras S (2009). Reply to ‘On the risk of extracting relevant information from random data’. J. Neural Eng..

[70] Yuan Z, Zhang D, Yang Y, Chen J, Li Y (2023). PPi: pretraining brain signal model for patient-independent seizure detection.

[71] Liu R (2021). Drop, swap, and generate: a self-supervised approach for generating neural activity.

[72] Schneider S, Lee J H, Mathis M W (2023). Learnable latent embeddings for joint behavioural and neural analysis. Nature.

[73] Pereira T D (2022). SLEAP: a deep learning system for multi-animal pose tracking. Nat. Methods.

[74] Dunn T W (2021). Geometric deep learning enables 3D kinematic profiling across species and environments. Nat. Methods.

[75] Jiang W B, Zhao L M, Lu B L (2024). Large brain model for learning generic representations with tremendous EEG data in BCI.

[76] Tu T (2024). Towards generalist biomedical AI. NEJM AI.

[77] Rudin C, Carlson D (2019). The secrets of machine learning: ten things you wish you had known earlier to be more effective at data analysis. Operations Research & Management Science in the Age of Analytics.

[78] Rudin C (2019). Stop explaining black box machine learning models for high stakes decisions and use interpretable models instead. Nat. Mach. Intell..

[79] Rudin C (2022). Why black box machine learning should be avoided for high-stakes decisions, in brief. Nat. Rev. Method Primers.

[80] Fellous J-M, Sapiro G, Rossi A, Mayberg H, Ferrante M (2019). Explainable artificial intelligence for neuroscience: behavioral neurostimulation. Front. Neurosci..

[81] Nori H, Jenkins S, Koch P, Caruana R (2019). InterpretML: a unified framework for machine learning interpretability.

[82] Struck A F (2017). Association of an electroencephalography-based risk score with seizure probability in hospitalized patients. JAMA Neurol.

[83] Kaur H (2020). Interpreting interpretability: understanding data scientists’ use of interpretability tools for machine learning.

[84] Gallagher N, Dzirasa K, Carlson D (2021). Directed spectrum measures improve latent network models of neural populations. https://proceedings.neurips.cc/paper/2021/hash/3d36c07721a0a5a96436d6c536a132ec-Abstract.html.

[85] Alagapan S (2023). Cingulate dynamics track depression recovery with deep brain stimulation. Nature.

[86] Hultman R (2018). Brain-wide electrical spatiotemporal dynamics encode depression vulnerability. Cell.

[87] Mague S D (2022). Brain-wide electrical dynamics encode individual appetitive social behavior. Neuron.

[88] Tozzi L (2024). Personalized brain circuit scores identify clinically distinct biotypes in depression and anxiety. Nat. Med..

[89] Trocellier D, N’Kaoua B, Lotte F (2024). Visual cues can bias EEG deep learning models.

[90] Dhar P (2020). The carbon impact of artificial intelligence. Nat. Mach. Intell..

[91] Grossman Y S (2022). Brain-wide oscillatory network selectively encodes aggression. bioxriv.

[92] Lotte F, Bougrain L, Cichocki A, Clerc M, Congedo M, Rakotomamonjy A, Yger F (2018). A review of classification algorithms for EEG-based brain–computer interfaces: a 10 year update. J. Neural Eng..

[93] Lotte F (2015). Signal processing approaches to minimize or suppress calibration time in oscillatory activity-based brain-computer interfaces. Proc. IEEE.

[94] Caliskan A, Bryson J J, Narayanan A (2017). Semantics derived automatically from language corpora contain human-like biases. Science.

[95] Webb E K, Etter J A, Kwasa J A (2022). Addressing racial and phenotypic bias in human neuroscience methods. Nat. Neurosci..

[96] Wassenaar E B, Van den Brand J G H (2005). Reliability of near-infrared spectroscopy in people with dark skin pigmentation. J. Clin. Monit. Comput..

[97] Caton S, Haas C (2023). Fairness in machine learning: a survey. ACM Comput. Surv..

[98] Castelnovo A, Crupi R, Greco G, Regoli D, Penco I G, Cosentini A C (2022). A clarification of the nuances in the fairness metrics landscape. Sci. Rep..

[99] Farahany N A (2023). The Battle for Your Brain : Defending the Right to Think Freely in the Age of Neurotechnology.

[100] Ramos K M (2019). NeuroView the NIH BRAIN Initiative: Integrating Neuroethics and Neuroscience.

[101] Dwork C, Roth A (2013). The algorithmic foundations of differential privacy. Found. Trends.

[102] Chambers D A, Feero W G, Khoury M J (2016). Convergence of implementation science, precision medicine, and the learning health care system: a new model for biomedical research. JAMA.

[103] Lin D (2020). The TRUST principles for digital repositories. Sci. Data.

